# Type 1 diabetes and diet-induced obesity predispose C57BL/6J mice to PM_2.5_-induced lung injury: a comparative study

**DOI:** 10.1186/s12989-023-00526-w

**Published:** 2023-04-17

**Authors:** Shen Chen, Miao Li, Rui Zhang, Lizhu Ye, Yue Jiang, Xinhang Jiang, Hui Peng, Ziwei Wang, Zhanyu Guo, Liping Chen, Rong Zhang, Yujie Niu, Michael Aschner, Daochuan Li, Wen Chen

**Affiliations:** 1 Guangdong Provincial Key Laboratory of Food, Nutrition and Health, Department of Toxicology, School of Public Health, Sun Yat-sen University, Guangzhou, 510080 China; 2 Department of Toxicology, School of Public Health, Hebei Medical University, Shijiazhuang, 050017 China; 3 Department of Molecular Pharmacology, Albert Einstein College of Medicine, Forchheimer 209, 1300 Morris Park Avenue, Bronx, NY 10461 USA

**Keywords:** Type 1 diabetes, High-fat diet, Particulate matter, Lung injury, RNA sequencing, Xenobiotic metabolism

## Abstract

**Background:**

Pre-existing metabolic diseases may predispose individuals to particulate matter (PM)-induced adverse health effects. However, the differences in susceptibility of various metabolic diseases to PM-induced lung injury and their underlying mechanisms have yet to be fully elucidated.

**Results:**

Type 1 diabetes (T1D) murine models were constructed by streptozotocin injection, while diet-induced obesity (DIO) models were generated by feeding 45% high-fat diet 6 weeks prior to and throughout the experiment. Mice were subjected to real-ambient PM exposure in Shijiazhuang City, China for 4 weeks at a mean PM_2.5_ concentration of 95.77 µg/m^3^. Lung and systemic injury were assessed, and the underlying mechanisms were explored through transcriptomics analysis. Compared with normal diet (ND)-fed mice, T1D mice exhibited severe hyperglycemia with a blood glucose of 350 mg/dL, while DIO mice displayed moderate obesity and marked dyslipidemia with a slightly elevated blood glucose of 180 mg/dL. T1D and DIO mice were susceptible to PM-induced lung injury, manifested by inflammatory changes such as interstitial neutrophil infiltration and alveolar septal thickening. Notably, the acute lung injury scores of T1D and DIO mice were higher by 79.57% and 48.47%, respectively, than that of ND-fed mice. Lung transcriptome analysis revealed that increased susceptibility to PM exposure was associated with perturbations in multiple pathways including glucose and lipid metabolism, inflammatory responses, oxidative stress, cellular senescence, and tissue remodeling. Functional experiments confirmed that changes in biomarkers of macrophage (F4/80), lipid peroxidation (4-HNE), cellular senescence (SA-β-gal), and airway repair (CCSP) were most pronounced in the lungs of PM-exposed T1D mice. Furthermore, pathways associated with xenobiotic metabolism showed metabolic state- and tissue-specific perturbation patterns. Upon PM exposure, activation of nuclear receptor (NR) pathways and inhibition of the glutathione (GSH)-mediated detoxification pathway were evident in the lungs of T1D mice, and a significant upregulation of NR pathways was present in the livers of T1D mice.

**Conclusions:**

These differences might contribute to differential susceptibility to PM exposure between T1D and DIO mice. These findings provide new insights into the health risk assessment of PM exposure in populations with metabolic diseases.

**Supplementary Information:**

The online version contains supplementary material available at 10.1186/s12989-023-00526-w.

## Background

Air pollution, especially ambient particulate matter (PM) pollution, is the fourth leading risk factor accounting for premature deaths and disability globally [1]. Long-term PM exposure is associated with multiple adverse health outcomes, including chronic respiratory and cardiovascular diseases [2, 3]. Despite the progressive decline in PM concentrations in China, nearly 80% of the population remains exposed to PM_2.5_ at concentrations that exceed China’s Air Quality Standards of 35 µg/m^3^ [4]. Moreover, the overall disease burden attributable to PM pollution is expected to continue to rise in the coming decades [5, 6]. Therefore, it’s essential to address the mechanisms underlying PM-induced adverse health effects and develop novel mechanism-based interventions.

The risks of PM-induced health effects can be modified both by extrinsic or intrinsic factors, such as dietary lifestyles and pre-existing metabolic disorders [7]. Largely due to substantial changes in dietary patterns (e.g., high-fat or high-calorie diets), the prevalence of overweight and obesity in Chinese adults is estimated to be 34.3% and 16.4%, respectively, which greatly contributes to the development of metabolic diseases [8]. Diabetes, classified into type 1 diabetes (T1D) and type 2 diabetes (T2D), is estimated to have affected 537 million adults worldwide in 2021 [9, 10]. Although T1D accounts for only 5–10% of diabetic patients, it has become one of the most common chronic diseases in childhood [9]. The incidence rate of T1D has been rising over the last decades, most likely due to environmental changes, such as air pollution [11, 12]. In contrast, T2D accounts for over 90% of diabetic patients, and is considered to be strongly related to overweight and obesity [9]. A large number of people with metabolic diseases are exposed to air pollution, facing a potentially higher risk of PM-related adverse health effects. Epidemiological studies suggest that metabolic disorders (e.g., obesity and diabetes) might be related to decreased lung function and increased respiratory and stroke-related mortality caused by air pollution [13–15]. However, it is unclear whether perturbations of signaling pathways triggered by distinct metabolic disorders have analogous effects in modifying PM-induced toxicity.

Although a number of animal studies have shown that PM exposure may exacerbate metabolic abnormalities [16–18], it remains unclear how pre-existing metabolic disorders affect PM-induced lung injury. T1D mice intratracheally instilled with diesel exhaust particles developed more severe lung inflammation and airway resistance [19]. Diet-induced obesity (DIO) led to enhanced lung injuries attributable to PM exposure [20]. Notably, metabolic diseases and dietary carbohydrate and fat composition have been associated with multiple adverse health effects of the respiratory system [21, 22]. However, it remains unclear whether altered glucose and lipid metabolism inherent to T1D or DIO have distinct effects on the lung microenvironment and susceptibility to PM exposure. Therefore, comparative studies under different metabolic states are critical to investigate the predisposition of PM-induced lung injury and its underlying mechanisms.

Xenobiotic metabolism plays a pivotal role in the transformation and disposition of environmental agents [23]. The responses to PM-bound chemicals retained in the respiratory tract or translocated to the systemic circulation largely depend on phase I/II metabolizing enzymes and transporters regulated by nuclear receptors (NRs) in lung or liver tissues [24–26]. However, the expression and activity of NRs are likely affected by various factors, such as pre-existing disease states, dietary patterns, and tissue specificity [27]. Particularly, peroxisome proliferator-activated receptors (PPARs) and liver X receptors (LXRs) are important transcriptional regulators in lipid and glucose metabolism, and their dysregulation has been implicated in the pathophysiology of metabolic syndrome [28]. Constitutive androstane receptor (CAR) activation and aryl hydrocarbon receptor (AHR) deficiency protected mice from HFD-induced obesity [29, 30]. Differential patterns in alterations of metabolizing enzymes and transporters have been revealed in multiple models of metabolic disorders, including ob/ob mice, T1D mice, and DIO mice [31–33]. Taken together, we hypothesized that distinct perturbation patterns of xenobiotic metabolism in response to PM exposure may play an important role in defining the health risks of PM exposure under different metabolic states.

Previously, we have constructed a whole-body exposure system to replicate real-ambient PM exposure scenarios for experimental animals, which simulates the natural state of PM exposure in humans to a great extent [34]. In the present study, we generated mouse models of T1D and DIO and subjected them to 4-week real-ambient PM exposure. Pulmonary and systemic injuries were assessed and compared between these two states of metabolic disorders. In addition, we elucidated the potential mechanisms with transcriptome analysis in lung and liver tissues, with a special emphasis on several molecular and xenobiotic metabolism pathways. These novel findings might lead to precise prevention and control of PM-related diseases.

## Results

### Establishment of mouse models with different patterns of metabolic disorders and PM exposure system

**Fig. 1:**
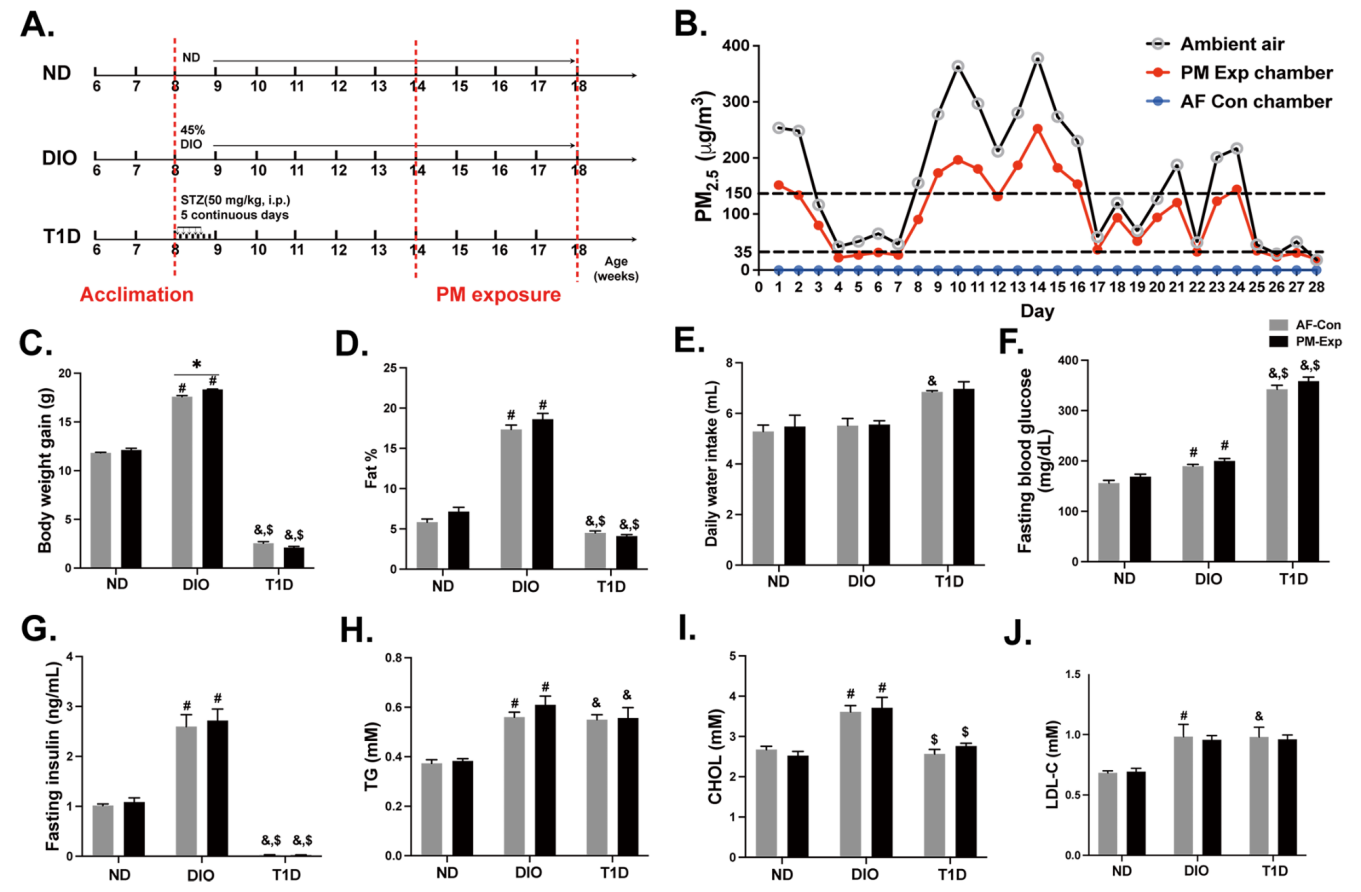
**Establishment of DIO and T1D mouse models for real-ambient PM exposure.** A schematic diagram of experimental design: six-week-old male C57BL/6 mice were injected with 50 mg/kg of streptozotocin (STZ) for 5 consecutive days or fed a 45% high-fat diet to generate DIO and T1D mice, followed by a 4-week real-ambient PM exposure (*n =* 20). **B.** The mean concentration of daily PM_2.5_ in ambient air (black line), PM exposure chambers (red line), and AF control chambers (blue line) during the exposure period, with reference to 35 µg/m^3^ of China’s Air Quality Standards and 150 µg/m^3^ of the threshold for heavy PM_2.5_ pollution in China. Eight representative indices of metabolic disorders that were divided into 4 categories: (1) body composition: body weight gain (**C**) and fat percentage (**D**); (2) daily water intake (**E**) (*n* = 10); (3) dysregulation of glucose metabolism: fasting glucose (**F**) and insulin levels (**G**) (*n =* 5); and (4) dysregulation of lipid metabolism: triglyceride (**H**), cholesterol (**I**), and low-density lipoprotein cholesterol or LDL-C (**J**) (*n* = 3). The data are presented as mean ± SEM. ^*^P < 0.05 (PM vs. AF); ^#^*P* < 0.05 (DIO vs. ND); ^&^*P* < 0.05 (T1D vs. ND); ^$^*P* < 0.05 (T1D vs. DIO).

PM exposure was performed in a real-ambient PM exposure system for 4 weeks from Jan 4th to Feb 1st, 2018, in Shijiazhuang City, China. The size of PM in the exposure chamber ranged from 0.5 to 1.5 μm with a mean diameter of 1.05 μm (Figure S1). The daily concentrations of PM_2.5_ in ambient air were well correlated with those in the exposure chamber over the course of 4-week exposure (Fig. 1B). The mean concentration of PM_2.5_ in the exposure chamber was 95.77 µg/m^3^. Accordingly, the cumulative lung burden of PM_2.5_ exposure was estimated at 31.32 µg/mouse using the Multiple-Path Particle Dosimetry Model software. Notably, PM_2.5_ was undetectable in the control chambers equipped with 3-layer air filters. The chemical composition of ambient PM_2.5_ was analyzed previously [35]. In brief, several PM-bound organic components (e.g., 16 US EPA priority polycyclic aromatic hydrocarbons) were quantified, some of which exceeded China’s Air Quality Standards, such as benzo[a]pyrene (10.13 vs. 2.5 ng/m^3^). Collectively, the PM exposure system in this study recapitulated the real-world scenario of heavily polluted areas in China.

To simulate different metabolic states in humans, we generated models of type I diabetes (T1D) and high-fat diet-induced obesity (DIO) in male C57BL/6J mice (Fig. 1A). T1D mice developed typical clinical manifestations of T1D, including severe hyperglycemia (approximately 350 mg/dL), insulin deficiency, weight loss, and polydipsia (Fig. 1C-G, Figures S2-S4). The body weight gain in DIO mice fed with a 45% high-fat diet for 10 weeks was 18.39% greater than in mice fed a normal diet (ND), consistent with moderate rodent obesity as reported previously [36]. Other changes in fat-related body composition, including fat mass, ingWAT, epiWAT, and iBAT, also supported the moderate obesity phenotype in DIO mice (Fig. 1C and D, Figure S2). In addition, DIO mice exhibited slightly elevated fasting blood glucose and 1.50-fold higher fasting insulin content (Fig. 1F and G, Figure S4). Based on plasma biochemical analysis, DIO mice displayed more severe dyslipidemia, with higher contents of triglycerides and cholesterol compared to T1D mice. The increase in LDL-C and the decrease in HDL-C also indicated lipid dysregulation in these two models (Fig. 1H-J, Figure S5 A). Moreover, more serious liver injuries appeared in T1D mice, manifested by higher ALT and AST levels (Figures S5 B and C). The increases in total protein, albumin, globulin, and creatinine in both mouse models implicated the presence of renal injury (Figures S5 D-I). Thus, we successfully generated two murine models with distinct patterns of metabolic abnormalities.

### The aggravating effects of T1D and DIO on PM-induced pulmonary and extra-pulmonary injury

**Fig. 2:**
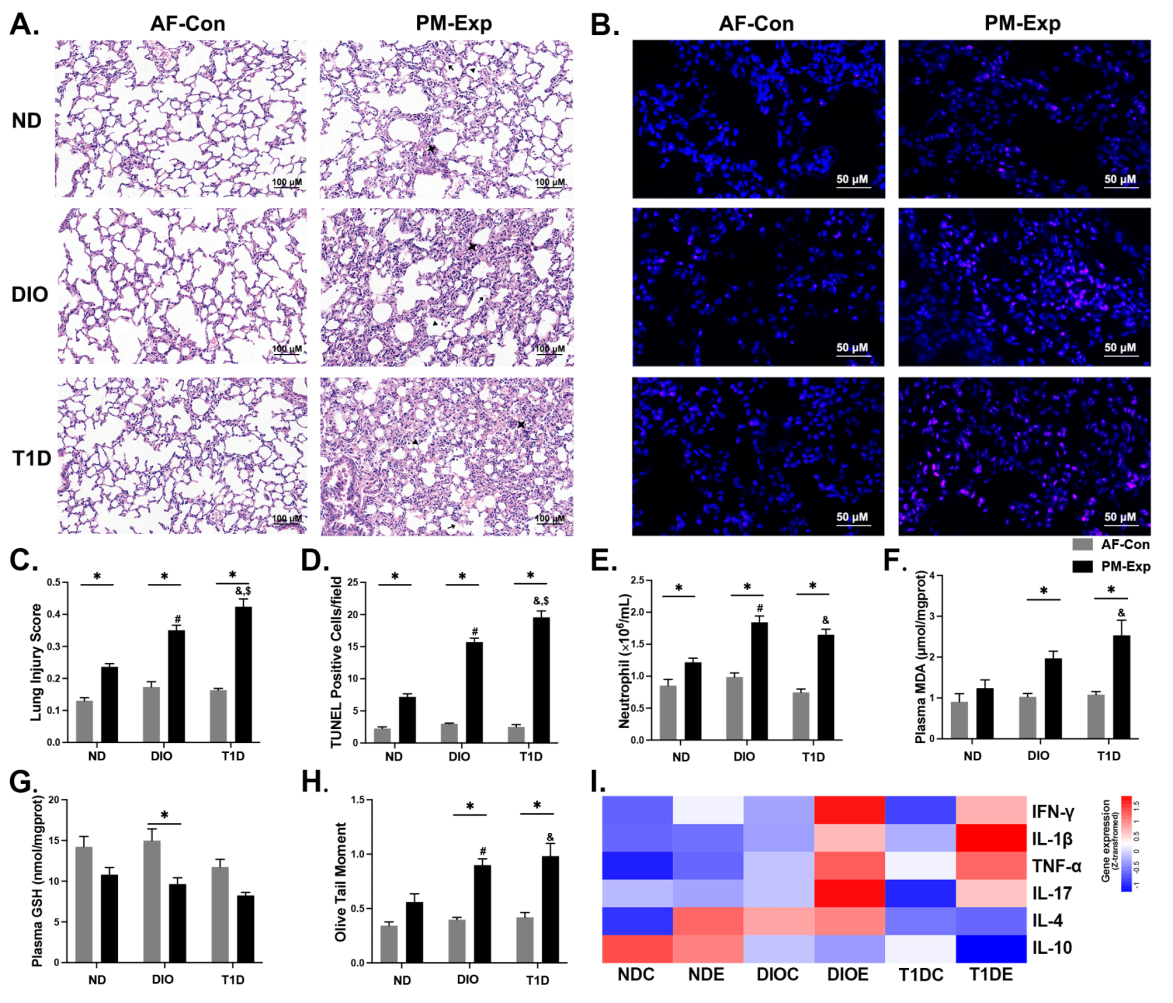
**T1D and DIO led to an enhanced PM-induced lung injury and systemic toxicity. (A)** Representative H&E-stained images (scale bar, 100 μm) in different mouse models with or without PM exposure, with typical pathological changes marked, including neutrophil infiltration in the interstitial space (✦) or in the alveolar space (▲), and proteinaceous debris in the airspace (↖). **(B)** Representative images of TUNEL staining (scale bar, 50 μm), positive cells were marked as red fluorescent. **(C)** Acute lung injury (ALI) scores derived from Fig. 2A. **(D)** Quantitative analysis of cell apoptosis based on Fig. 2B. **(E)** The number of neutrophils in the peripheral blood (*n* = 10). Systemic oxidative stress was indicated by the content of plasma MDA (**F**) and plasma GSH (**G**). **H.** DNA damage indicated by quantitative analysis of olive tail moment from the Comet Assay. **I.** The heatmap based on plasma cytokines levels (IFN-γ, IL-1β, TNF-α, IL-17, IL-4, and IL-10) (All *n* = 5). The data are presented as mean ± SEM. ^*^P < 0.05 (PM vs. AF); ^#^*P* < 0.05 (DIO vs. ND); ^&^*P* < 0.05 (T1D vs. ND); ^$^*P* < 0.05 (T1D vs. DIO).

Male DIO, T1D, and ND-fed mice (*n* = 20) were exposed to real-ambient PM or filtered air (AF) for 4 weeks. Lung histopathological examination showed that PM exposure led to interstitial neutrophil infiltration, alveolar septal thickening, and sporadic proteinaceous debris in the airspace (Fig. 2A). Notably, these manifestations were more significant in DIO and T1D mice, and the latter exhibited the most severe inflammation. The acute lung injury (ALI) scores showed an increase of 48.47% and 79.57% in DIO and T1D mice, respectively, compared with ND-fed mice upon PM exposure (Fig. 2C). Lung coefficients were increased in DIO and T1D mice upon PM exposure, while no changes appeared in ND-fed mice (Figure S6). Moreover, DIO and T1D led to a 1.20- and 1.73-fold increase in the number of apoptotic cells in the lungs upon PM exposure compared with ND-fed mice (Fig. 2B and D). In agreement with these pathological observations, the total cell number, LDH, total protein, and albumin in bronchoalveolar lavage fluid (BALF) were more significantly increased in T1D mice than in DIO mice (Figures S7 A-D). These results demonstrate that metabolic disorders may predispose mice to severe pulmonary injury upon PM exposure, particularly in T1D mice.

To examine the extra-pulmonary injury induced by PM exposure, we quantified systemic inflammation by blood cell counting and plasma cytokine analysis. Compared with PM-exposed ND-fed mice, DIO and T1D led to an increased neutrophil count by 51.44% and 35.58%, respectively (Fig. 2E). Similar alterations were also observed for WBC and lymphocyte counts (Figures S7 E and F). Oxidative stress was assessed by plasma MDA, urine 8-OHdG, and plasma GSH. Plasma MDA was upregulated by 58.87% or 104.19% in DIO or T1D mice upon PM exposure compared with ND-fed mice (Fig. 2F), consistent with the changes in plasma GSH and urinary 8-OHdG (Fig. 2G, Figure S7 G).

Genotoxicity was detected by the Comet assay and indicated by the olive tail moment (OTM). Although there were no significant changes in ND-fed mice, 1.26- and 1.36-fold increases in OTM were observed in DIO and T1D mice following PM exposure, respectively (Fig. 2H). Increased pro-inflammatory cytokines (IFN-γ, IL-1β, TNF-α, and IL-17) appeared in two mouse models but were more pronounced in DIO mice upon PM exposure (Fig. 2I). These observations contrast the extent of systematic injury in the two metabolic states in response to PM exposure, consistent with pulmonary pathological findings. Particularly, DIO tends to promote systemic inflammation and T1D predominantly enhances oxidative stress and genetic damage.

### Comparisons of pathway perturbations in lungs of T1D and DIO mice

To decipher the regulatory mechanisms underlying PM-induced lung injury under different metabolic states, we performed lung transcriptomics analysis to identify pulmonary differentially expressed genes (DEGs) (Figure S8). Both principal component analysis (PCA) and hierarchical clustering analysis (HCA) showed clear distinctions among groups under different metabolic states (Figures S9 A and B). Next, we utilized Ingenuity Pathway Analysis (IPA) software to address the biological relevance of DEGs. The Circo heatmaps illustrated the top 30 enriched pathways from comparisons among groups. The comparisons between AF and PM groups revealed the effects of PM on pulmonary injury (Figures S9 C and D). Notably, the patterns of disease susceptibility and pathway perturbations attributed to PM exposure varied greatly among the three different metabolic states.

**Fig. 3:**
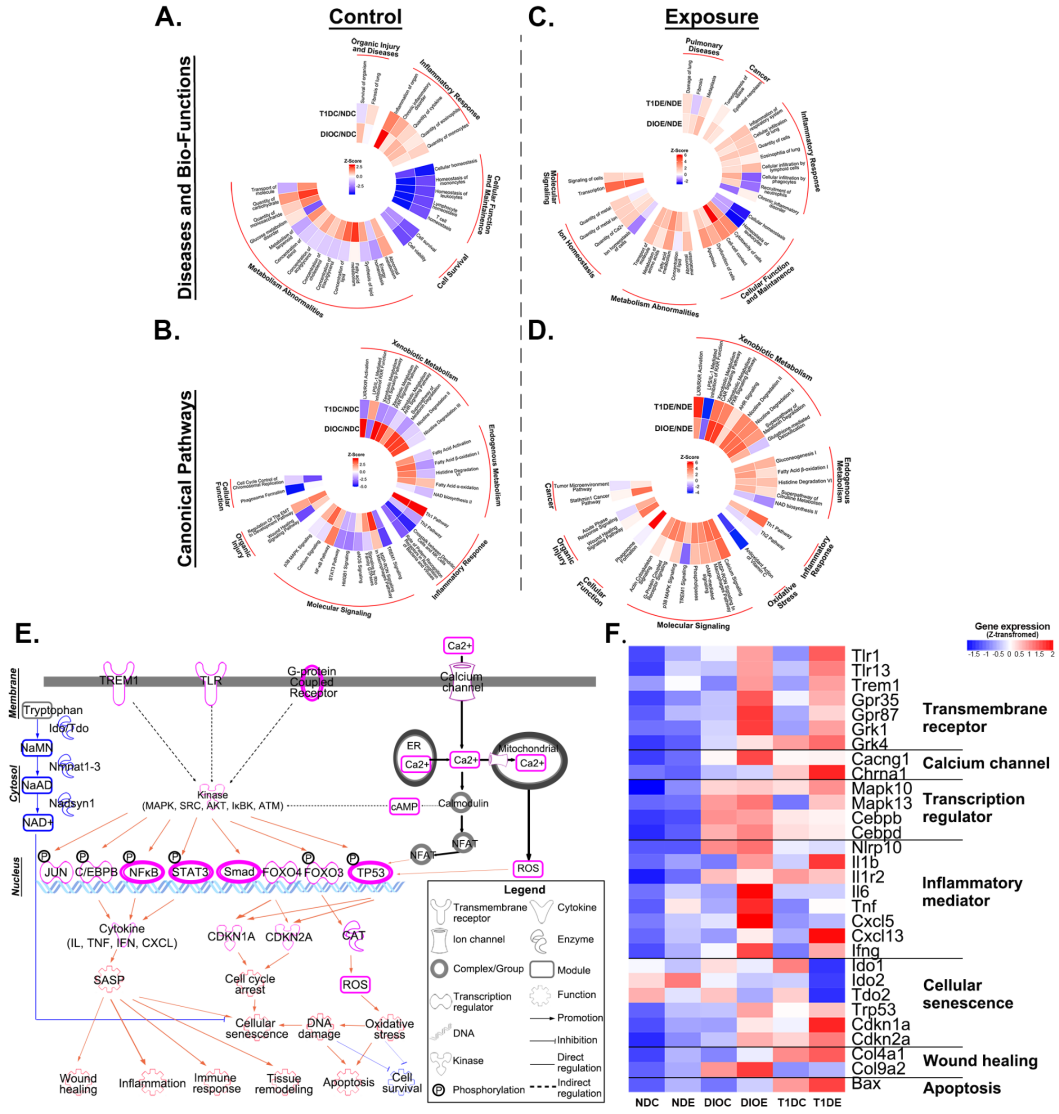
**Perturbation of molecular pathways in lungs of DIO and T1D mice exposed to PM.** Mouse lungs were subjected to RNA sequencing to delineate biological mechanisms underlying increased susceptibility to PM-induced lung damage. DEGs obtained between DIO or T1D and ND comparisons without or with PM exposure were analyzed by IPA software for Disease and Biological Function annotation (**A**, **C**) and Canonical Pathway analysis (**B**, **D**). The results were shown as the Circo heatmaps consisting of the top 30 enriched pathways. **E.** Molecular regulatory network by which DIO and T1D aggravated PM-induced lung injury was illustrated derived from the transcriptome results. Pink elements and yellow arrows indicate the upregulation in DIO or T1D mice compared with ND-fed mice, while blue elements and arrows indicate the downregulation changes. **F.** Key genes involved in enhanced lung injury (Fig. 3E) were confirmed at the mRNA level and shown as a heatmap (*n* = 3)

To explore whether and how different metabolic states affected pulmonary injury to PM exposure, we carried out comprehensive analyses among T1D, DIO, and ND-fed mice (Fig. 3A and B). First, we sought to determine the molecular basis of increased susceptibility to PM exposure by comparisons between T1D or DIO and ND mice in AF control groups (TIDC or DIOC vs. NDC, C signifies AF control). As shown in Fig. 3A, metabolic abnormalities were the most prominent features (14/30 top enriched pathways) but varied greatly between these two metabolic states. Glucose metabolism was consistently disrupted in both states, while lipid metabolism was upregulated in DIOC mice, yet slightly downregulated in T1DC mice, consistent with the metabolic characteristics of these animal models. With regard to disease-related features, such as inflammatory response, cellular functions, and organic injury, we found similar patterns of metabolic disruption in both murine models. Interestingly, the activity of perturbated xenobiotic metabolism pathways was upregulated in the lungs of DIOC mice, but downregulated in T1DC mice (Fig. 3B), intimating that DIO mice might possess enhanced metabolic activity towards environmental chemicals.

Next, we identified critical pathway perturbations involved in aggravating pulmonary injury upon PM exposure by comparative analysis among T1DE, DIOE, and NDE mice (E signifies PM exposure) (Fig. 3C and D). The disease and biological function analysis revealed that PM exposure may result in a variety of disease outcomes, not limited to metabolic abnormalities (Fig. 3C). Specifically, T1D mice exhibited greater effects on cellular functions, ion homeostasis, and lung damage, while DIO mice showed enhanced macrophage infiltration, neutrophil recruitment, fibrosis, and cancer. Significant perturbations attributable to PM were also revealed through canonical pathway analysis. It was obvious that the magnitude of pathway activation was more pronounced in T1D mice (Fig. 3D). Inflammation-related pathways (e.g., macrophage-stimulating protein (MSP)-RON, mitogen-activated protein kinase (MAPK)) were profoundly altered due to PM exposure. Pathways related to macrophage activation and polarization (Th1/Th2, MSP-RON) showed similar perturbations in the two murine models. However, phagosome formation (an important step in phagocytosis) was upregulated in response to PM, particularly in DIOE mice. ROS production pathway was activated while the antioxidant action of vitamin C was severely inhibited following PM exposure in both groups of mice. In addition, the wound healing pathway related to tissue repair was inhibited in T1D mice but activated in DIO mice. Accordingly, the regulatory network by which T1D or DIO aggravates PM-induced lung injury was derived from lung transcriptome profiles (Fig. 3E), and the representative genes were confirmed at the mRNA level (Fig. 3F). The key molecular events were corresponding to inflammatory responses, oxidative stress, cellular senescence, tissue remodeling, etc. Taken together, lung transcriptomics revealed that T1D and DIO play important roles in the regulation of glucose and lipid metabolism and contribute to increased susceptibility to PM exposure.

### Key molecular events involved in the enhanced lung response to PM exposure in T1D and DIO mice

**Fig. 4:**
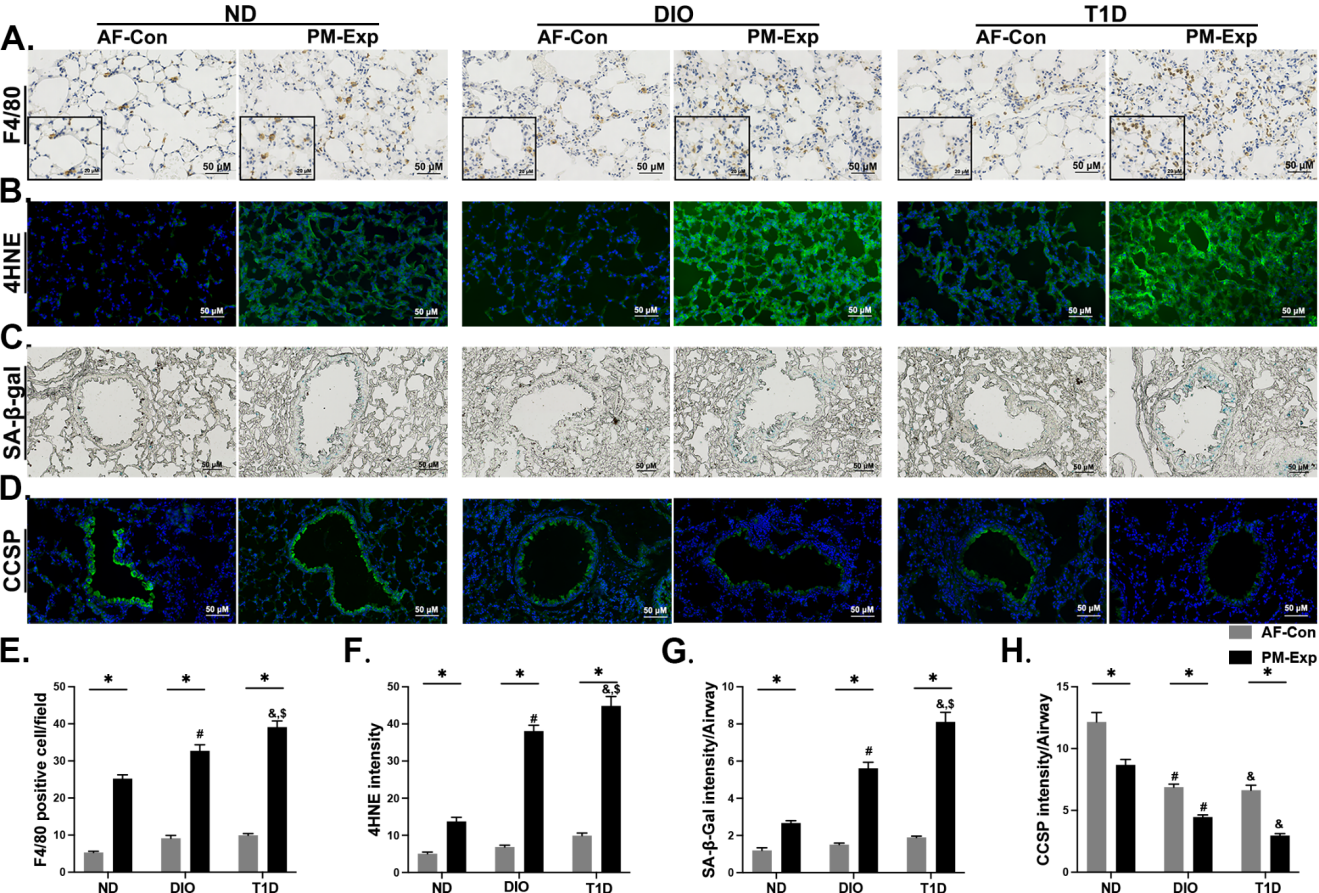
**The effects of PM exposure on the key molecular events in the lungs of DIO and T1D mice.** Representative images of F4/80 immunochemical staining (scale bar, 50 μm) (**A**) and corresponding quantitative results (**D**). Representative images of 4HNE immunofluorescence staining (scale bar, 50 μm) (**B**) and corresponding quantitative results (**F**). Representative images of SA-β-gal staining (scale bar, 50 μm) (**E**) and corresponding quantitative results (**G**). Representative images of CCSP immunofluorescence staining (scale bar, 50 μm) (**D**) and corresponding quantitative results (**H**) (All *n* = 5). The data are presented as mean ± SEM. ^*^P < 0.05 (PM vs. AF); ^#^*P* < 0.05 (DIO vs. ND); ^&^*P* < 0.05 (T1D vs. ND); ^$^*P* < 0.05 (T1D vs. DIO).

Next, we determined the critical events involved in aggravating PM-induced lung injury in the lungs of the two mouse models. Lung inflammation was characterized by the recruitment and polarization of macrophages, inflammatory cell count analysis, and cytokine quantification in BALF. Macrophage recruitment (F4/80+) upon PM exposure was elevated by 29.78% and 55.04% in DIO and T1D mice, respectively, compared with ND-fed mice (Fig. 4A and E). Macrophage count in BALF showed analogous results, while the increases in polymorphonuclear leukocytes (PMNs) were more pronounced in DIO mice (Figures S10 A and B). We further examined the polarization of M1 and M2 macrophages in BALF, which indicated the pro- and anti-inflammatory effects, respectively. Although the increase in M1 and decrease in M2 counts were inherent to both murine models, a larger M1/M2 ratio was observed in DIO mice (Figures S10 C-E). The secretion of pro-inflammatory cytokines (IFN-γ, IL-1β, TNF-α, and IL-17) in BALF were significantly increased in both mouse models. However, the anti-inflammatory effects of IL-4 and IL-10 in DIO and T1D mice were suppressed compared with ND-fed mice upon PM exposure, particularly more significant in T1D mice (Figure S10 F). To assess the extent of oxidative stress, we measured the levels of lipid peroxidation products, 4-HNE and MDA. Compared with ND-fed mice, 4-HNE was significantly increased in both groups of mice upon PM exposure with 2.26 or 1.77 times higher in T1D or DIO mice compared with their corresponding AF control mice (Fig. 4B and F). Consistently, the changes in MDA showed analogous quantitative relationships (Figure S10 G). Collectively, lung inflammatory responses upon PM exposure varied greatly between T1D and DIO mice, while oxidative damage was consistently more pronounced in T1D mice.

Another crucial molecular event is cellular senescence resulting from dysregulated NAD biosynthesis, cell cycle arrest, and DNA damage (Fig. 3E). DIO and T1D led to SA-β-gal accumulation by a 1.09- and 2.04-fold increase in response to PM exposure, respectively (Fig. 4C and G). Clara cell secretory protein (CCSP) is a marker of Clara cells that are linked to cell senescence and airway repair capacity [37]. Compared with ND-fed mice, the content of CCSP in lung tissues was considerably reduced by 48.58% and 65.81% in DIO and T1D mice, respectively, upon PM exposure (Fig. 4D and H). Decreased CCSP cytokine levels were also observed in BALF, and lower in T1D mice (Figure S10 H). Taken together, we demonstrate that disorder of multiple molecular events, including lung inflammatory responses, oxidative stress, cellular senescence, and reduced tissue repair capacity in both metabolically abnormal mice might contribute to PM exposure-induced adverse health effects.

### Differential perturbation patterns of xenobiotic metabolism in T1D and DIO mice

**Fig. 5:**
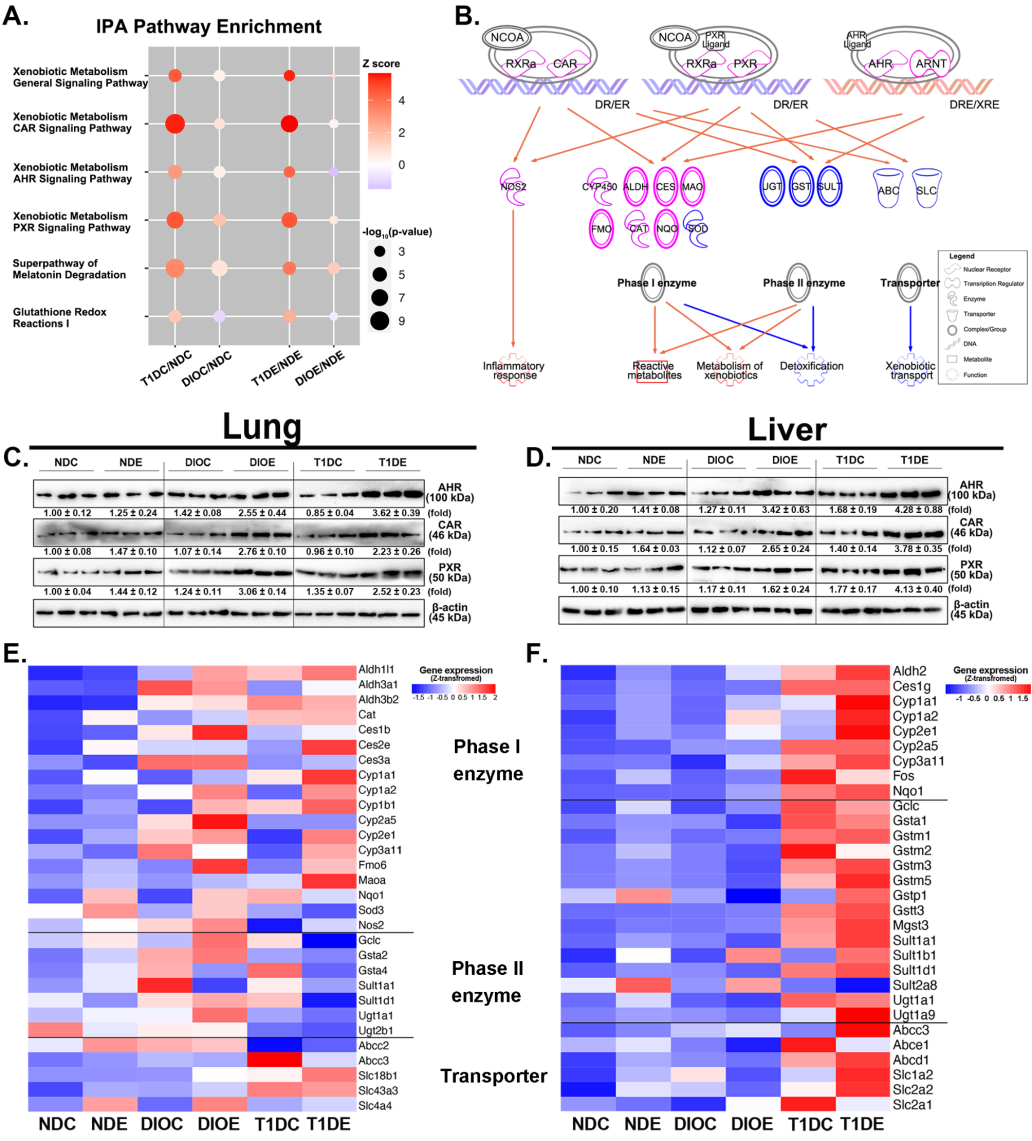
**Differential xenobiotic metabolism patterns in the lungs and livers of DIO and T1D mice. (A)** Perturbations of key xenobiotic metabolism pathways before and after PM exposure in the livers of DIO or T1D mice compared with ND-fed mice. **(B)** The diagram presented dysregulated xenobiotic metabolism in lung tissues, exemplified by PM-induced changes in T1D mice compared to ND-fed mice. Pink elements and yellow arrows indicate the upregulation in T1D mice compared with ND-fed mice, while blue elements and arrows indicate the downregulation changes. Immunoblotting of AHR, CAR, and PXR in the lung (**C**) and liver (**D**). The data are presented as mean ± SEM. The heatmap showed the mRNA levels of phase I/II metabolizing enzymes and transporters in the lung (**E**) and liver (**F**). The z-score transformation was utilized to calculate the relative level of gene expression. (All *n* = 3)

Xenobiotic metabolism plays a pivotal role in the biotransformation and disposal of PM. We speculate that a comprehensive analysis of xenobiotic metabolism between DIO and T1D mice may explain why or how different metabolic disorders predispose mice to PM exposure. In the lungs, NR signaling pathways (CAR, AHR, LXR, and pregnane X receptor (PXR)) remained active in DIO mice with or without PM exposure, while the activity of NR pathways transformed from inhibition to activation in response to PM exposure in T1D mice (Fig. 3B and D). Moreover, the GSH-mediated detoxification pathway upon PM exposure was activated in DIO mice but inactivated in T1D mice (Fig. 3D). Liver RNA-seq was also performed to identify the disruptions of xenobiotic metabolism-related pathways (Figures S11 and 12). The activation of NR pathways was more significant in the livers of T1D mice, while the detoxification pathway was consistently inhibited in those of DIO mice but activated in those of T1D mice (Fig. 5A). These results reveal that pathways related to xenobiotic metabolism in the lungs or livers exhibit tissue- and metabolic state-specific perturbation patterns in T1D and DIO mice.

**Fig. 6:**
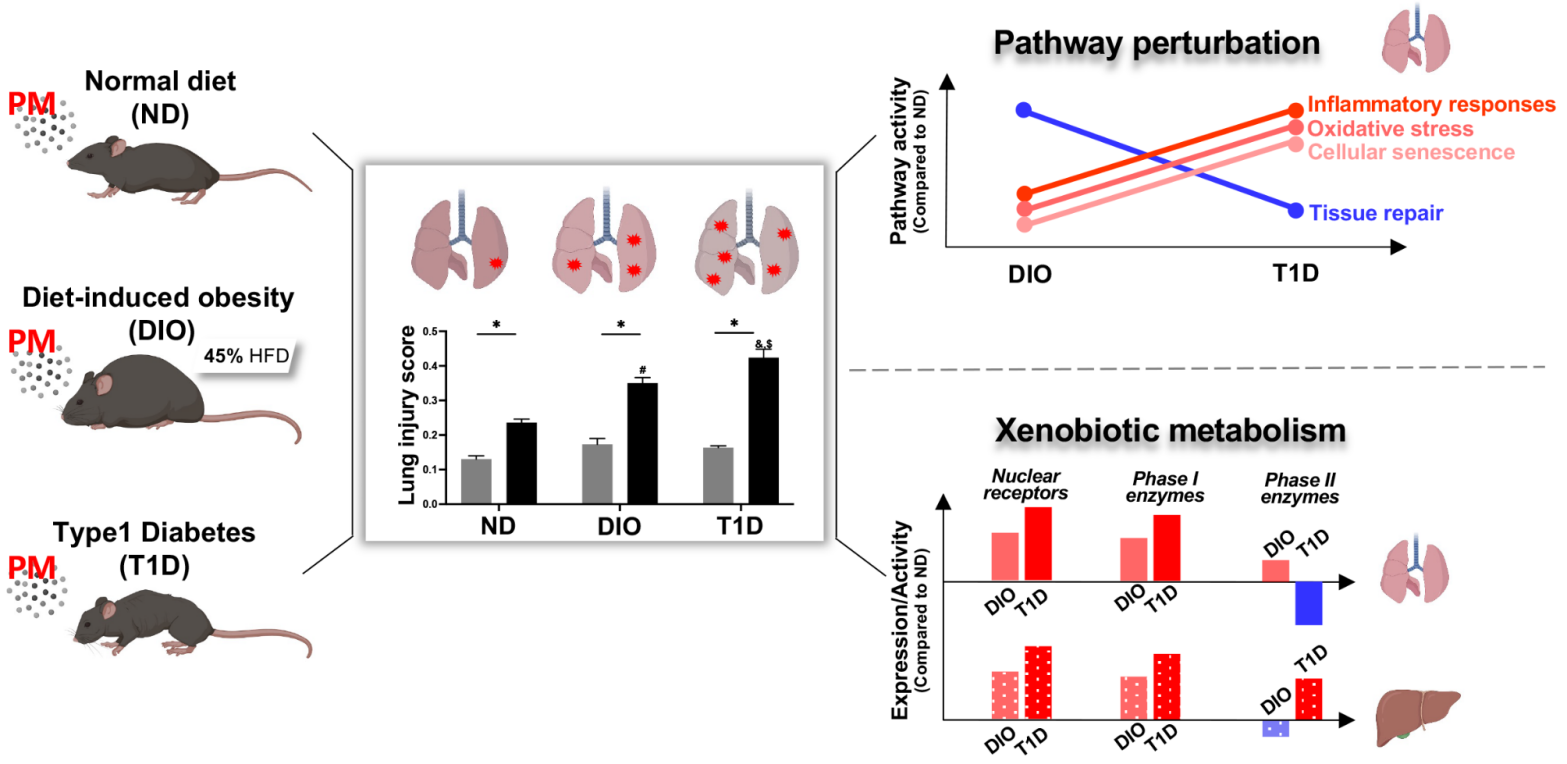
**A summary diagram illustrates the differential susceptibility to PM-induced lung injury in T1D, DIO, and ND-fed mice.**

Next, we constructed regulatory networks of xenobiotic metabolism, exemplified by alterations in T1D mouse lungs upon PM exposure (Fig. 5B). The expression of three key NRs (AHR, CAR, and PXR) was confirmed at the protein level, which was concordant with the upregulation of corresponding NR pathways in the lungs and livers of T1D and DIO mice (Fig. 5C and D. Figure S13). mRNA expressions and activities of key xenobiotic enzymes were examined in lungs and livers. In the lungs, both DIO and T1D mice displayed an increase in most phase I enzymes upon PM exposure, e.g., CYP450s (Fig. 5E, Figures S14 A-C). However, decreased expression of phase II enzymes was observed only in the lungs of T1D mice upon PM exposure (Fig. 5E), consistent with the decline in SOD, GST, and GSH contents in T1D mice compared with DIO mice (Figures S14 D-F). In the livers, increases in the majority of the surveyed metabolizing enzymes and transporters appeared only in T1D mice with or without PM exposure (Fig. 5F, Figures S14 G-L). Interestingly, a reduction in the expression of several phase II enzymes was present in DIO mice, including GSTs and UDP-glucuronosyltransferases (UGTs), and several transporter subtypes (Fig. 5F). These changes were further corroborated by the degree of enzyme activity of SOD and GST, which were lower by 18.52% and 28.67% in DIO vs. T1D mice (Figures S14 J and K). Taken together, compared to DIO mice, upregulation in NR pathways and downregulation in the GSH-mediated detoxification pathway were more pronounced in the lungs of T1D mice, while a more significant upregulation of NR pathways was present in the livers of T1D mice. These pattern-specific differences in xenobiotic metabolism between T1D and DIO mice are likely responsible for the distinct susceptibility to PM-induced toxic effects.

## Discussion

Unhealthy dietary lifestyles and pre-existing metabolic diseases serve as potential risk factors for increasing the risks of adverse health effects induced by PM exposure [7]. Although epidemiological studies have linked obesity and diabetes to the exacerbation of respiratory diseases from air pollution [13–15], the underlying mechanisms remain largely unknown. In this study, murine models of DIO and T1D were generated and subjected to real-ambient PM exposure. Although both DIO and T1D mice were more susceptible to PM exposure, more severe lung injury appeared in T1D mice. Lung transcriptome analysis revealed distinct pathway perturbations in the regulation of glucose and lipid metabolism, inflammatory responses, oxidative stress, cellular senescence, and tissue remodeling. Analysis of xenobiotic metabolism-related pathways further identified differential regulatory patterns in DIO and T1D mice, which might underlie the regulatory mechanisms of PM-induced adverse health effects (Fig. 6). These findings shed light on how T1D and DIO increase susceptibility to PM-induced lung injury in mice through different regulatory modes of metabolism.

Dietary lifestyles and disease conditions may disturb organismal homeostasis, which confers vulnerability to environmental stimuli. T1D is caused by autoimmune-mediated pancreatic β-cell destruction, resulting in absolute insulin deficiency. The onset of T1D is thought to be the interaction of polygenic and environmental triggers [9]. In contrast, T2D is usually considered to be associated with unhealthy lifestyles, such as high-fat diets [9]. T1D has become one of the most common chronic diseases in children and adolescents, while T2D is the most common type of diabetes [10]. This study employed T1D and DIO (may progress to T2D) mouse models, which differed widely in many aspects [10]. Generally, both models share a common feature of hyperglycemia, but DIO has an additional characteristic of dyslipidemia [38]. The deeply branched vascularization makes the lung vulnerable to hyperglycemia [21], while lipid-rich pulmonary surfactants are readily altered by dyslipidemia [22], thereby disrupting lung homeostasis [39]. Therefore, DIO and T1D lead to distinct lung injuries, creating unfavorable lung microenvironments that may modify PM disposition and toxicity, possibly through different biological mechanisms. In the present study, hyperglycemia was observed in both metabolically abnormal mice, particularly in T1D mice, while dyslipidemia was more pronounced in DIO mice. These metabolic characteristics were further supported by lung RNA-seq results, providing evidence that abnormal metabolic states may result in disruption of lung homeostasis. Conceivably, the states of T1D or DIO may confer mice increased susceptibility to PM exposure.

Previous studies have demonstrated that PM exposure can exacerbate metabolic abnormalities in DIO and T1D models, such as potentiation to adipose inflammatory responses, cardiac fibrosis, hepatic fibrosis, and vascular endothelial dysfunction [16–18, 40–44]. By contrast, less attention has been paid to the molecular mechanisms by which different metabolic diseases affect PM-induced lung toxicity. In addition, less evidence provided from comparative evidence impedes the protection of high-risk subpopulations under PM exposure. Here, we revealed that the states of DIO or T1D resulted in an enhanced lung injury towards PM exposure, indicated by pathological changes, immune cell infiltration, changes in epithelial permeability, and apoptosis. Intriguingly, T1D mice presented more pronounced lung injury upon PM exposure. Lung transcriptome analysis demonstrated that increased susceptibility to PM exposure in these two metabolically abnormal mice was closely associated with inflammatory responses, oxidative stress, cellular senescence, and reduced tissue repair capacity.

Inflammatory responses and oxidative stress underlie the lung’s defense response to PM [3]. It has been demonstrated that HFD-induced obesity predisposes individuals to lung inflammation, lipid peroxidation, airway hyperresponsiveness, and the development of chronic respiratory diseases [45]. T1D led to pulmonary artery hyperresponsiveness via oxidative stress and cyclooxygenase-2 induction [46]. Moreover, DIO and T1D have been reported to result in greater pulmonary responses to multiple stimuli (e.g., cigarette smoke, inorganic arsenic, and diesel exhaust particles) [47–49]. In this study, it’s noteworthy that T1D consistently exerted more significant alterations than DIO upon PM exposure. The differences between DIO and T1D regarding the pathways associated with inflammatory responses (TREM1, PRR, MAPK, HMGB1, STAT3, Th1/Th2, and MSP-RON) and oxidative stress (production of NO and ROS, the antioxidant action of vitamin C) might be responsible for enhanced lung toxicity of PM exposure in T1D mice.

Prior studies have revealed that DNA damage and cellular senescence are involved in PM-induced adverse health effects and implicated pulmonary fibrosis in STZ-induced diabetic rodents [50, 51]. More recently, HFD has been found to exacerbate aging-induced lung injury and delay airway repair by disturbing the function of AT2 cells and bronchioalveolar stem cells [52, 53], suggesting a potential role of lung cell senescence in response to environmental stress under adverse metabolic states. In this study, pathway perturbations were linked to DNA damage, cellular senescence, tissue repair, and wound healing in T1D and DIO mice upon PM exposure, but more pronounced in T1D mice. The evidence enhances the notion that the abnormal metabolic state of diabetes leads to greater health risk than hyperlipidemia towards PM exposure.

Sufficient evidence has proved that inhaled particles can be directly translocated into the circulation and accumulated in other target organs, such as the heart, liver, and brain [54, 55]. Subsequently, PM-bound chemicals provoke a series of extra-pulmonary effects often manifested as cardiovascular and metabolic effects [56–60], which are linked to systemic inflammation, genotoxicity, oxidative stress, apoptosis, etc. [61]. LPS-induced pulmonary inflammation enhanced the extra-pulmonary translocation of PM [62], indicating that induction of inflammatory mediators may also promote PM translocation, thereby exerting stronger systemic toxicity. Moreover, HFD resulted in more significant changes in circulatory inflammatory mediators upon PM exposure [44], while T1D enhanced systemic inflammation and oxidative stress from diesel exhaust particle exposure [40]. In the present study, DIO led to increased secretion of pro-inflammatory cytokines in the plasma, suggesting the occurrence of systemic chronic low-grade inflammation [63]. However, more pronounced disturbances in oxidative stress and genotoxicity prevailed in T1D mice exposed to PM. Overall, DIO and T1D promote PM-mediated systemic effects on different molecular toxicological consequences.

Xenobiotic metabolism has received great attention due to its dominant role in the metabolism of PM-bound chemicals [64]. Dietary lifestyles and disease conditions affect the activity of metabolizing enzymes by interacting with xenobiotic response elements (XRE), resulting in alterations in cellular response. HFD-induced obesity modulated the expression and activity of hepatic metabolizing enzymes and transporters [31, 32]. T1D induced increased expressions of CAR-dependent hepatic CYP450s in mice [33]. In addition, preferential expression and distribution of metabolizing enzymes and transporters in different tissues confer tissue-selective chemical toxicity [65]. It has been reported that co-exposure to HFD and cigarette smoke altered CYP450s in lung and liver tissues to varying degrees [66], implicating that the interplay between metabolism and environmental stimuli is critical in the determination of health effects. In this study, metabolic activation and detoxification of PM-bound chemicals in the lungs and livers were associated with multiple pathways of xenobiotic metabolism, including several NR signaling pathways and the GSH-mediated detoxification pathway. The metabolic state- and tissue-specific modulatory patterns were well correlated with the regulatory functions of NRs in T1D and DIO mice. As the main effectors of xenobiotic metabolism, increased phase I enzymes but decreased phase II enzymes appeared in the lungs of T1D mice, which might contribute to more persistent and severe lung injury. Taken together, these findings reveal the potential mechanisms by which two adverse metabolic states affect the disposition and toxicity of xenobiotics.

Epidemiological studies have indicated that metabolic diseases modified the effect of air pollution on lung function loss and respiratory mortality [13, 15]. A recent study reported the application of metabolic syndrome biomarkers in the prediction of the development of lung injury in the World Trade Center cohort, suggesting that systemic inflammation contributes to lung function impairment [14]. In this study, we compared the differential susceptibility to PM exposure, providing evidence for policymakers to develop population-specific targets for the prevention or treatment of air pollution-induced respiratory diseases. For example, the upregulation of oxidative stress and cellular senescence pathways in both mice suggests a potentially protective effect of antioxidants or senolytics. Notably, the glutathione-mediated detoxification pathway was significantly suppressed only in T1D mice. Since exogenous supplementation with GlyNAC (a combination of glycine and N-acetylcysteine) could efficiently increase the content of cellular glutathione [67], it’s worth exploring whether it could act as a powerful means against PM-induced lung injury in diabetic populations. Previously, we demonstrated the protective effect of calorie restriction on PM-induced toxic effects in mice, which might be due to enhanced xenobiotic metabolism and detoxification [35]. Although it is evident that abnormal metabolic states accelerated functional impairments in murine exposed to PM, perturbations of specific pathways derived from transcriptomic analysis need to be cautiously interpreted and population-based studies are required to verify the potential cause-effect relationship.

## Conclusions

In summary, the present study demonstrates that the state of diabetes (T1D) and hyperlipemia (DIO) aggravate lung injury upon real-ambient PM exposure. Particularly, T1D exerts greater effects on PM-induced lung injury, which are closely associated with perturbations of glucose and lipid metabolism, inflammatory responses, oxidative stress, cellular senescence, and tissue repair pathways. Moreover, differential perturbations in xenobiotic metabolism pathways might further contribute to a higher risk of PM-related lung injury in T1D mice. These findings provide strong evidence of how different metabolic states impact adverse health effects induced by PM exposure.

## Methods

### Animal models

Six-week-old male C57BL/6J mice were purchased from the Model Animal Research Center of Nanjing University (Nanjing, Jiangsu, China). One hundred and twenty (120) mice were randomly divided into three groups to generate models of type 1 diabetes (T1D, *n* = 40), diet-induced obesity (DIO, *n* = 40), and normal diet-fed mice (ND, *n* = 40). After a 2-week acclimation, 50 mg/kg of streptozotocin (STZ) was intraperitoneally injected for 5 consecutive days to induce T1D in mice fed with a normal diet. STZ can selectively destruct pancreatic islet β-cells and repeated low doses of STZ injection have been commonly used in the induction of T1D murine models [68]. DIO mice were given *ad libitum* access to a 45% high-fat diet (HFD) for 6 weeks prior to and throughout 4-week PM exposure (D12451; Research Diets, Inc., New Brunswick, NJ, USA) (Fig. 1A). 45% HFD is considered to be more relevant to human physiology than 60% formula in rodent studies [69] and 10-week feeding of 45% HFD can generate mouse models with moderate obesity [36]. ND-fed mice were fed a standard rodent diet (AIN93G purified diet; Guangdong Medical Laboratory Animal Center, Guangzhou, China). Energy percentages of macronutrients and formulation of these two diets were listed in Table S1-3. Food pellets and water bottles were weighed weekly, and the average daily food intake and water consumption were calculated weekly. All animal procedures and experiments were approved by the Animal Care and Use Committee of the Model Animal Research Center of Sun Yat-sen University and Hebei Medical University.

### Real-ambient particulate matter exposure

Whole-body PM inhalational exposure was conducted in a real-ambient PM exposure system located in Shijiazhuang City, China. The characteristics of the system were described in our previous study [34]. Briefly, the individually ventilated caging (IVC) system was remodeled with three-layer HEPA filters to block PM_2.5_. HEPA-filtered air was imported into the air filter (AF) chambers (control group), while ambient air was introduced directly into the PM exposure chambers.

Following 6-week different diet feeding, mouse models (age of 14 weeks) were successfully established. Based on their body weight, mouse models were matched into 20 pairs in each group, and randomly divided into six subgroups, ND-AF, ND-PM, DIO-AF, DIO-PM, T1D-AF, and T1D-PM (*n* = 20). Mice (5 mice/cage) were housed in solid-bottom caging with corn cob bedding with *ad libitum* access to food and water under 12-h daylight/darkness cycle. Mice were subjected to PM exposure 24 h per day and 7 days per week for 4 weeks, from Jan 4th to Feb 1st, 2018. With the advantage of simulating the natural state and around-the-clock PM exposure, this IVC-based exposure system enables us to observe the health effects of sustained high PM exposure and to recapitulate real scenarios of human exposure to a great extent.

PM_2.5_ concentrations in the chambers were monitored using an Aerosol Detector DUSTTRAKTM II and the particle size spectrum was analyzed using an Aerodynamic Particle Sizer Spectrometer 3321 (TSI Incorporated, Shoreview, MN, USA). The meteorological conditions inside the chambers were closely monitored to maintain a relatively constant state (Table S4). The cumulative lung burden of PM exposure estimated using the Multiple-Path Particle Dosimetry Model was reported previously [35]. At the end of the PM exposure, mice were euthanized and the biological samples were collected for further analysis.

### Body composition measurement

Whole-body composition parameters (*n* = 10) of mice, including total body fat mass and lean mass, were measured using an EchoMRI™ Body Composition Analyzer (Echo Medical System, Houston, TX). Each mouse without anesthesia was placed in an appropriate-size holder. The holder was then inserted into a tubular space in the system, followed by a whole-body scan. As a result, the parameters were recorded as grams.

### Examination of blood glucose and insulin levels

Mice were fasted for 12 h before examination of fasting glucose and insulin levels (*n* = 5). Blood samples from mice aged 8 weeks to 18 weeks were collected from the tail vein for the detection of glucose levels using an Accu-Chek Aviva blood glucose meter (Roche, Basel, Switzerland). Fasting insulin levels were examined with a Mouse Insulin ELISA Detection Kit (Mercodia, Uppsala, Sweden) according to the manufacturer’s instructions. The absorbance of samples was measured by a BioTek 800 TS Absorbance Reader (BioTek Instruments, Inc., USA).

### Estimation of cumulative lung burden of PM_2.5_ exposure

The cumulative lung burden of PM_2.5_ exposure was calculated using the following equation:

$$\begin{array}{c}{\rm{Estimated}}\,{\rm{cumulative}}\,{\rm{lung}}\,{\rm{burden = MV \times T \times }}\\{\rm{ CON \times DF}}\end{array}$$

where MV is the minute ventilation volume in the exposed mice (mL/min); T is the total exposure time (min); CON is the mean concentration of PM_2.5_ (µg/m^3^); DF is the pulmonary deposition fraction of PM_2.5_ (m^3^), which is estimated by the Multiple-Path Particle Dosimetry Model software (MPPD 3.04) via https://www.ara.com/products/multiple-path-particle-dosimetry-model-mppd-v-304) [70].

### Tissue preparation and histopathological analysis

Several types of tissues, including lung, liver, heart, kidney, spleen, epididymal and inguinal white adipose tissues (epiWAT and ingWAT), and interscapular brown adipose tissues (iBAT) were collected and weighed, followed by 24-h fixation in 4% paraformaldehyde (PFA) at room temperature. Five-micrometer of paraffin-embedded lung and liver tissues were mounted on slides and subjected to hematoxylin and eosin (H&E) staining and immunohistochemical and immunofluorescence examinations. The histological analysis of acute lung injury (ALI) was scored as described previously [71].

### Hematological and biochemical analysis

At the end of the experiment, mice were anesthetized with 100 mg/kg of pentobarbital sodium before being sacrificed. Peripheral blood was exsanguinated from the inferior vena cava and collected into an EDTA-coated tube (BD Biosciences, San Jose, California). The hematological analysis (*n* = 10) was conducted by an automatic hematology analyzer (HemaVet 950FS, DREW, USA). The plasma was isolated from the remaining blood by centrifugation at 450×g at room temperature for 10 min. An aliquot of 100 µL plasma was used for biochemical analysis (*n* = 3) using an automatic biochemical analyzer Pointcare M3 (MNCHIP, Tianjin, China).

### BALF analysis

The cells and supernatant in bronchoalveolar lavage fluid (BALF) (*n* = 5) were separated by centrifugation at 400×g for 7 min at 4 °C. The contents of total protein (TP), lactate dehydrogenase (LDH), and albumin (ALB) in BALF supernatant were determined by BCA Protein Assay Kit (Beyotime Biotech Inc., Nantong, China), LDH Release Assay Kit (Promega Corporation, Madison, WI, USA), and Albumin Assay Kit (Nanjing Jiancheng Bioengineering Inc., China), respectively. The number of cells was determined with a cell counter (Beckman, Coulter, CA, USA). 1 × 10^4^ cells were spread on a microscope slide, fixed with 96% ethanol, and stained with May-Grünwald-Giemsa. The number of macrophages and polymorphonuclear neutrophils (PMNs) was counted under a light microscope (Leica, Germany).

### TUNEL staining

Five-micrometer lung sections were stained with TUNEL Apoptosis Assay Kit (Beyotime Biotech Inc., Nantong, China) to quantify cell apoptosis according to the manufacturer’s instructions. Murine lung sections absent in any stimuli were incubated with 0.01 U/µL DNase I for 10 min at room temperature and regarded as a positive control. To quantify apoptotic cells, 20 random fields per section in each group were counted under a light microscope (Leica, Germany), and the average number of apoptotic cells per section was calculated. *n* = 5.

### Senescence-associated beta-galactosidase analysis

Five-micrometer frozen lung sections were stained with Senescence β-Galactosidase Staining Kit (Solarbio Life Sciences, Beijing, China) according to the manufacturer’s instructions. To quantify senescent cells, 20 random fields per section in each group were counted under a light microscope (Leica, Germany), and the average number of senescent cells per section was calculated. *n* = 5.

### Immunofluorescence and immunohistochemistry analysis

4-hydroxynonenal (4-HNE) is a well-studied aldehyde product of phospholipid peroxidation, indicating the extent of oxidative stress. Clara cell secretory protein (CCSP) is a marker of Clara cells. Both 4-HNE and CCSP measurements were performed by immunofluorescence staining. Briefly, lung tissue slides were deparaffinized, rehydrated, and heated in 0.1 M citrate buffer (pH 5.8) for antigen retrieval. After blocking with 2% BSA for 30 min at room temperature, the slides were incubated with primary antibodies against 4-HNE (#ab48506; Abcam, Cambridge, UK; 1:300) or CCSP (#ab213203; Abcam, Cambridge, UK; 1:2000) overnight at 4°C, followed by Alexa Fluor 488-conjugated IgG second antibody (Thermo Fisher Scientific™, Waltham, MA, USA; 1:1000) and 4’,6-diamidino-2-phenylindole (DAPI) for 1 h at room temperature in the dark. Sections were then observed under a laser scanning confocal microscope (Leica, Germany). F4/80, a marker of mouse mature macrophages, was detected with immunohistochemistry staining as described previously [35]. All *n* = 5.

### Flow cytometry analysis

1 × 10^6^ cells isolated from BALF were blocked with anti-mouse FcR antibody (CD16/CD32; Biolegend, USA) at 4 °C for 15 min in FACS buffer (PBS with 2% FBS and 1 mM EDTA). Cells were stained with Zombie NIR Fixable Viability Kit at 4 °C for 15 min (Biolegend, USA) to discriminate dead cells, and then stained with antibodies for F4/80 (FITC; Biolegend, USA), CD11b (PerPC/Cy5.5; Biolegend, USA), CD11c (PE; Biolegend, USA), CD206 (APC; Biolegend, USA) or isotype control at 4 °C for 30 min. Cells were washed 3 times with FACS buffer, fixed with the Fixation/Permeabilization buffer at 4 °C for 40 min, and washed 3 times in Permeabilization buffer (eBioscience, USA). Cells were then resuspended in FACS buffer and analyzed by flow cytometry using the CytoFlex platform (Beckman Coulter, USA). All *n* = 5.

### Assessment of oxidative stress

The contents of malondialdehyde (MDA) in lung tissues, BALF, and plasma were measured by thiobarbituric acid (TBA) reactivity using the commercial colorimetric Lipid Peroxidation MDA Assay Kit (Beyotime Biotech Inc., Nantong, China). Glutathione-S-transferase (GST) activities in lung and liver tissues were examined using the GST Assay Kit (Solarbio Life Sciences, Beijing, China). The contents of reduced glutathione (GSH) in BALF, lung tissues, and liver tissues were examined using the GSH and GSSG Assay Kit (Beyotime Biotech Inc., Nantong, China). Activities of superoxide dismutase (SOD) were quantified using Total Superoxide Dismutase Assay Kit with WST-8 (Beyotime Biotech Inc., Nantong, China). To detect urinary 8-hydroxy-2’-deoxyguanosine (8-OHdG), 100 µg DNA was dissolved in 80 µL deionized water, with 5 µL of [^13^C_10_, ^15^N_5_]-8-OHdG at 740 µg/L adding to the DNA solution as an internal standard. The DNA samples were then converted into single-stranded DNA by incubation at 95 °C for 10 min and digested to single nucleotide fragments by treatment with 5 units of nuclease P1 at 37 °C for 1 h. The mixture was subsequently dephosphorylated by incubation with 10 units of alkaline phosphatase at 37 °C for 1 h. Following centrifugation at 15,000 g for 15 min, the supernatant was subjected to LC-MS/MS analysis. The concentration of cellular 8-OHdG was calculated by extrapolating the peak area of the sample from a set of standards (0, 0.25, 0.5, 0.75, 1.5, 2, 4 µg/L). Urinary 8-OHdG concentrations were normalized to urinary creatinine levels. All *n* = 3.

### Alkaline Comet assay

100 µL of blood was used for the Comet assay using the protocol reported previously [72]. Briefly, 5 µL of blood was mixed with 20 µL of 0.8% low melting point agarose at 37℃ and spread onto a CometAssay® HT 20-well slide (Trevigen). Alkaline lysis, gel electrophoresis, neutralization, and fixation were performed sequentially. Following PI staining, the slides were analyzed under a fluorescence microscope (Nikon Eclipse Ti-E), and 150 cells per slide were randomly selected and scored by Comet Assay Software Project-1.2.2 (University of Wroclaw, Poland). The index of olive tail moment (OTM) was used to indicates the degree of DNA damage. 16HBE cells exposed to UV radiation for 30 min were regarded as the positive control. *n* = 5.

### Cytokine analysis

Cytokines in BALF and plasma (*n* = 5), including interferon-gamma (IFN-γ), interleukin-1 beta (IL-1β), tumor necrosis factor-alpha (TNF-α), interleukin-17 (IL-17), interleukin-4 (IL-4), and interleukin-10 (IL-10) were measured by using enzyme linked immunosorbent assay (ELISA) Assay Kits (R&D Systems, MN, USA). The z-score transformation was utilized to calculate the relative level of cytokines according to the following equation: $Z=\frac{X-\stackrel{-}{X}}{SD}$ ($\stackrel{-}{X}$ is the mean value, SD is the standard deviation).

### Determination of enzymatic activity

Activities of cytochrome P450 enzymes including CYP1A1, CYP1B1, and CYP3A4 in lung and liver tissues, were measured by using P450-Glo™ CYP1A1, CYP1B1, and CYP3A4 Assay System (Promega Corporation, Madison, WI, USA), following the manufacturer’s instructions. All *n* = 5.

### Quantitative real-time polymerase chain reaction (qRT-PCR)

Total RNA in the lung or liver tissues was isolated using TRIzoL reagent (Invitrogen, Carlsbad, CA, USA), and reverse transcription was carried out by using an Advantage RT-for-PCR Kit (Takara, Tokyo, Japan). Next, the qRT-PCR was conducted using SYBR® Green Realtime PCR Master Mix (Toyobo, Tokyo, Japan) and analyzed in a Real-Time PCR System. β-actin is served as an internal control. The 2^−ΔΔCt^ method was used to calculate the relative expression of mRNAs. The primers used for qPCR are listed in Table S5. The heatmaps were generated by the R package Pheatmap using the z-score transformed values. The z-score transformation was utilized to calculate the relative level of transcripts according to the following equation:$Z=\frac{X-\stackrel{-}{X}}{SD}$. *n* = 3.

### Immunoblotting analysis

Lung and liver tissues were excised and homogenized, and the total cellular proteins were extracted by ice-cold RIPA lysis buffer (150 mmol/liter of NaCl, 1% Triton X-100, 0.5% deoxycholate, 0.1% SDS, and 50 mM Tris (pH 7.4)) containing protease inhibitors. The lysates were centrifuged at 12,000×g for 20 min at 4 °C. Soluble proteins (20 µg) were separated by 8 ~ 12% SDS-PAGE gel electrophoresis and immunoblotting was conducted with primary antibodies against AHR (#67,785; Proteintech Group, AL, USA; 1:3000), CAR (#ab186869; Abcam, Cambridge, UK; 1:1000), PXR (#67,912; Proteintech Group, AL, USA; 1:3000), and β-actin (#60,008; Proteintech Group, AL, USA; 1:4000), followed by HRP-conjugated secondary antibodies (Beyotime Biotech Inc., Nantong, China; 1:3000). Immunolabeling was visualized via incubating the bands with Western Blotting Chemiluminescence Luminol Reagent (Santa Cruz Biotechnology, CA, USA). The density of the specific protein bands was quantified using ImageJ software. *n* = 3.

### RNA sequencing and bioinformatics analysis

Lung and liver tissues isolated from 6 groups of mice (*n* = 3) were selected for RNA sequencing using the BGISEQ-500 sequencing technology platform. The raw reads were first filtered using the SOAPnuke software (https://github.com/BGI-flexlab/SOAPnuke). After quality control, the clean reads were aligned to a reference genome (version: GRCm38) using Bowtie2 (http://bowtie-bio.sourceforge.net/index.shtml), followed by quantification of transcript expression levels using the RSEM method (http://deweylab.biostat.wisc.edu/rsem/rsem-calculate-expression.html). Differentially expressed genes (DEGs) were identified with the R package DEGseq, with the fold change value between groups greater than 1.5 times and the adjusted *P*-value less than 0.05 as the threshold. A full list of DEGs was presented in Additional file 2. Partial least squares-discriminant analysis (PLS-DA) was performed using the R package ropls. Heatmaps were generated with the R package Pheatmap. To reveal the biological implications of DEGs between groups, the canonical pathway analysis and the disease and biological function analysis were carried out using Ingenuity Pathway Analysis (IPA) software (Qiagen, Germany). Significant differences were defined as the *P*-value was less than 0.01, and the absolute value of the z score was greater than 2. Based on biological pathways enriched from pairwise comparisons, the Circo heatmaps were generated by the R package circlize 0.4.14. All RNA-seq datasets have been uploaded to the GEO repository (accession number: GSE228200).

### Statistical analysis

The data are presented as mean ± standard error of mean (SEM). All statistical analysis was performed using SPSS 22.0 statistical software (SPSS Inc., IL, USA). Since data included two categorical independent variables, with or without PM exposure under three types of metabolic states (ND/T1D/DIO), we applied two-way analysis of variance (2-way ANOVA) followed by Bonferroni’s post hoc test to analyze differences among multiple groups. Differences were considered statistically significant at *P* < 0.05. All bar plots were drawn with Prism 9 (GraphPad Software Inc., California, USA).

## Electronic supplementary material

Below is the link to the electronic supplementary material.

Supplementary Material 1

Supplementary Material 2

## Data Availability

The datasets used and/or analysed during the current study are available from the corresponding author on reasonable request.

## List of abbreviations

PM_2.5_: Fine particulate matter
T1D: Type 1 diabetes
DIO: Diet-induced obesity
ALI: Acute lung injury
BALF: Bronchoalveolar lavage fluid
4HNE: 4-hydroxynonenal
SA-β-gal: Senescence-associated beta-galactosidase
CCSP: Clara cell secretory protein
8-OHdG: 8-hydroxy-2’-deoxyguanosine
OTM: Olive tail moment
NR: Nuclear receptor
CAR: Constitutive androstane receptor
AHR: Aryl hydrocarbon receptor
PXR: Pregnane X receptor
GSH: Glutathione
CYP450: Cytochrome P450
