# Supplementary material for: Type 1 diabetes and diet-induced obesity predispose C57BL/6J mice to PM2.5-induced lung injury: a comparative study
Source: Part Fibre Toxicol. 2023 Apr 17;20:10. doi: 10.1186/s12989-023-00526-w (PMC10108512; doi:10.1186/s12989-023-00526-w)
Supplement: Supplementary file 1 — Supplementary Material 1 [file 12989_2023_526_MOESM1_ESM.docx]

**Supplementary material**

**Type 1 diabetes and diet-induced obesity predispose C57BL/6J mice to PM_2.5_-induced lung injury: a comparative study**

Shen Chen^#1^, Miao Li^#1^, Rui Zhang^#1^, Lizhu Ye^1^, Yue Jiang^1^, Xinhang Jiang^1^, Hui Peng^1^, Ziwei Wang^1^, Zhanyu Guo^1^, Liping Chen^1^, Rong Zhang^2^, Yujie Niu^2^, Michael Aschner^3^, Daochuan Li^1^, Wen Chen^*1^

^1^Guangdong Provincial Key Laboratory of Food, Nutrition and Health, Department of Toxicology, School of Public Health, Sun Yat-sen University, Guangzhou 510080, China.

^2^Department of Toxicology, School of Public Health, Hebei Medical University, Shijiazhuang, 050017, China.

^3^Department of Molecular Pharmacology, Albert Einstein College of Medicine, Forchheimer 209, 1300 Morris Park Avenue, Bronx, NY, 10461, USA.

^#^These authors contributed equally: Shen Chen, Miao Li, Rui Zhang.

^*^Correspondence and requests for materials should be addressed to Wen Chen, Email: [chenwen@mail.sysu.edu.cn](mailto:chenwen@mail.sysu.edu.cn).

**Supplementary figures**

**
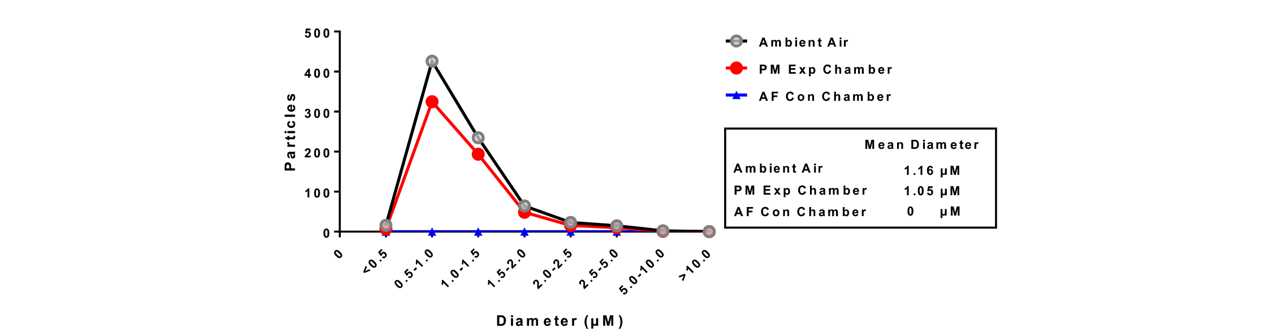
**

**Figure S1. The distribution of PM size.**

The distribution of particle diameters in PM samples from ambient air (black line with grey circles), the PM exposure chambers (blue line with blue triangles), and the AF control chambers (red line with red circles) were compared. In contrast to no particles detected in samples from AF control chambers, samples from ambient air and the PM exposure chambers were comparable in particle size, ranging from 0.5 to 1.5 micrometers.

**Figure S2. Body composition analysis.**

The dynamic changes in body weight (A), fat mass (B), lean mass (C), quadriceps (D), inguinal white adipose tissues (ingWAT) (E), epididymal white adipose tissues (epiWAT) (F), and interscapular brown adipose tissues (iBAT) (G) from age 8 weeks to 18 weeks in different mouse models (*n* = 10). The data are presented as mean ± SEM. ^*^*P* < 0.05 (PM vs. AF). ^#^*P* < 0.05 (DIO vs. ND). ^&^*P* < 0.05 (T1D vs. ND). ^$^*P* < 0.05 (T1D vs. DIO).

**Figure S3. The average daily water consumption and food intake.**

A. The weekly changes in the average daily water consumption. B. The weekly changes in the average daily food intake (left), and the average daily food intake at the 18-week of age (right). *n* = 10. The data are presented as mean ± SEM. ^*^*P* < 0.05 (PM vs. AF). ^#^*P* < 0.05 (DIO vs. ND). ^&^*P* < 0.05 (T1D vs. ND). ^$^*P* < 0.05 (T1D vs. DIO).


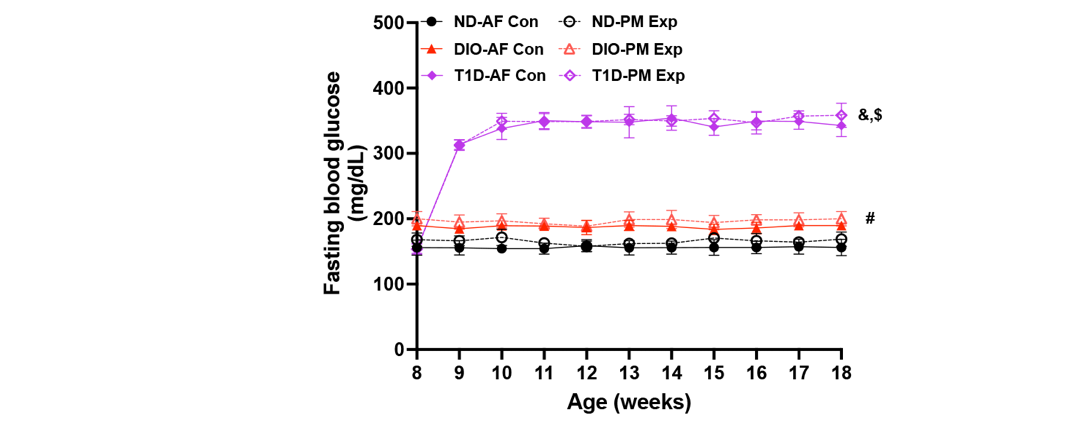


**Figure S4. Dynamic changes in fasting blood glucose levels in different mouse models from 8 to 18 weeks of age.**

After five consecutive injections of STZ, T1D mice began to present markedly elevated fasting blood glucose levels. DIO mice that were fed a 45% high-fat diet for 10 weeks had slightly higher glucose than mice fed with the ND regimen (*n* = 10). The data are presented as mean ± SEM. ^*^*P* < 0.05 (PM vs. AF). ^#^*P* < 0.05 (DIO vs. ND). ^&^*P* < 0.05 (T1D vs. ND). ^$^*P* < 0.05 (T1D vs. DIO).

**Figure S5. Plasma biochemical analysis in different mouse models upon PM exposure.**

The biochemical analysis was conducted in ND-fed (control), DIO, and T1D mice following 4-week PM exposure. A. Low-density lipoprotein cholesterol (LDL-C). B. Alanine transaminase (ALT). C. Aspartate transaminase (AST). D. Total protein (TP). E. Total bilirubin (TBIL). F. Albumin (ALB). G. Globulin (GLO). H. Albumin/Globulin (A/G). I. Creatinine (CRE). *n* = 3. The data are presented as mean ± SEM. ^*^*P* < 0.05 (PM vs. AF). ^#^*P* < 0.05 (DIO vs. ND). ^&^*P* < 0.05 (T1D vs. ND). ^$^*P* < 0.05 (T1D vs. DIO).

**Figure S6. Organ coefficients in different mouse models upon PM exposure.**

Organ coefficients were calculated in ND-fed, DIO, and T1D mice upon 4-week PM exposure. A. Lung coefficient. B. Kidney coefficient. C. Liver coefficient. D. Spleen coefficient. *n* = 10. The data are presented as mean ± SEM. ^*^*P* < 0.05 (PM vs. AF). ^#^*P* < 0.05 (DIO vs. ND). ^&^*P* < 0.05 (T1D vs. ND). ^$^*P* < 0.05 (T1D vs. DIO).

**
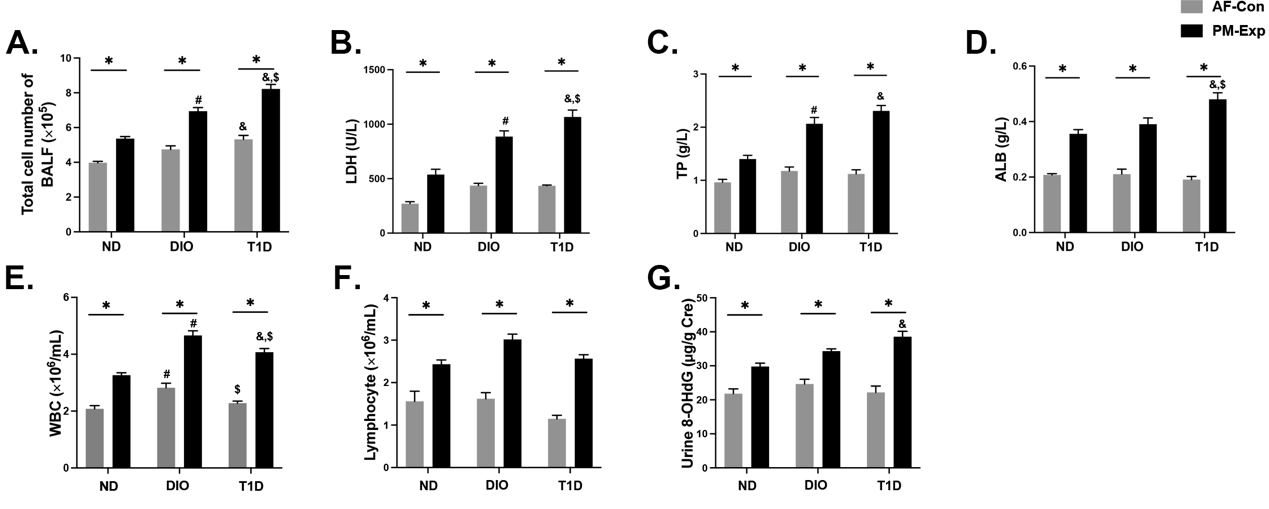
**

**Figure S7. The state of T1D and DIO led to enhanced lung injury and systemic toxicity upon PM exposure.**

A. Count of total cell number in BALF. The contents of lactase hydrogenase (B), total protein (C), and albumin (D) in BALF. Cell differential analysis showed the quantitative relationship between the count of WBCs (E) and lymphocytes (F). The content of urine 8-OHdG (G). *n* = 5. The data are presented as mean ± SEM. ^*^*P* < 0.05 (PM vs. AF). ^#^*P* < 0.05 (DIO vs. ND). ^&^*P* < 0.05 (T1D vs. ND). ^$^*P* < 0.05 (T1D vs. DIO).

**
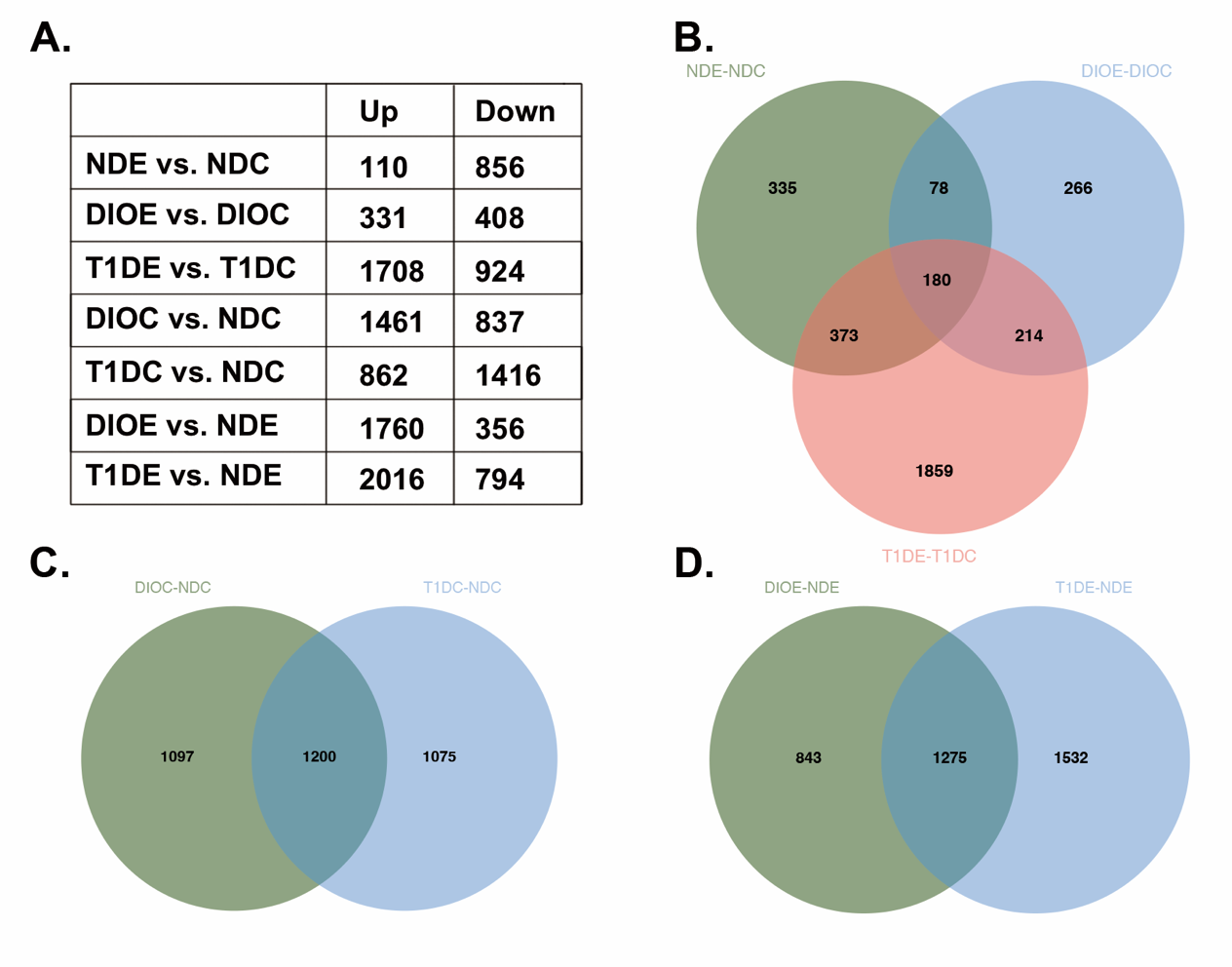
Figure S8. Multiple comparisons between differentially expressed genes (DEGs) in lung tissues.**

A. The table presents the number of DEGs (up- or down-regulated ), which were subjected to multiple comparisons. The Venn plots display the relationship among DEGs from multiple comparisons, including DEG _NDE vs. NDC_, DEG _T1DE vs. T1DC_, and DEG _DIOC vs. NDC_ (B), DEG _DIOC vs. NDC_ and DEG _T1DC vs. NDC_ (C), and DEGinvolved _DIOE vs. NDE_ and DEG _T1DE vs. NDE_ (D). *n* = 3. The threshold was set as the fold change (FC) value greater than 1.5 times, and the adjusted *P*-value less than 0.05.


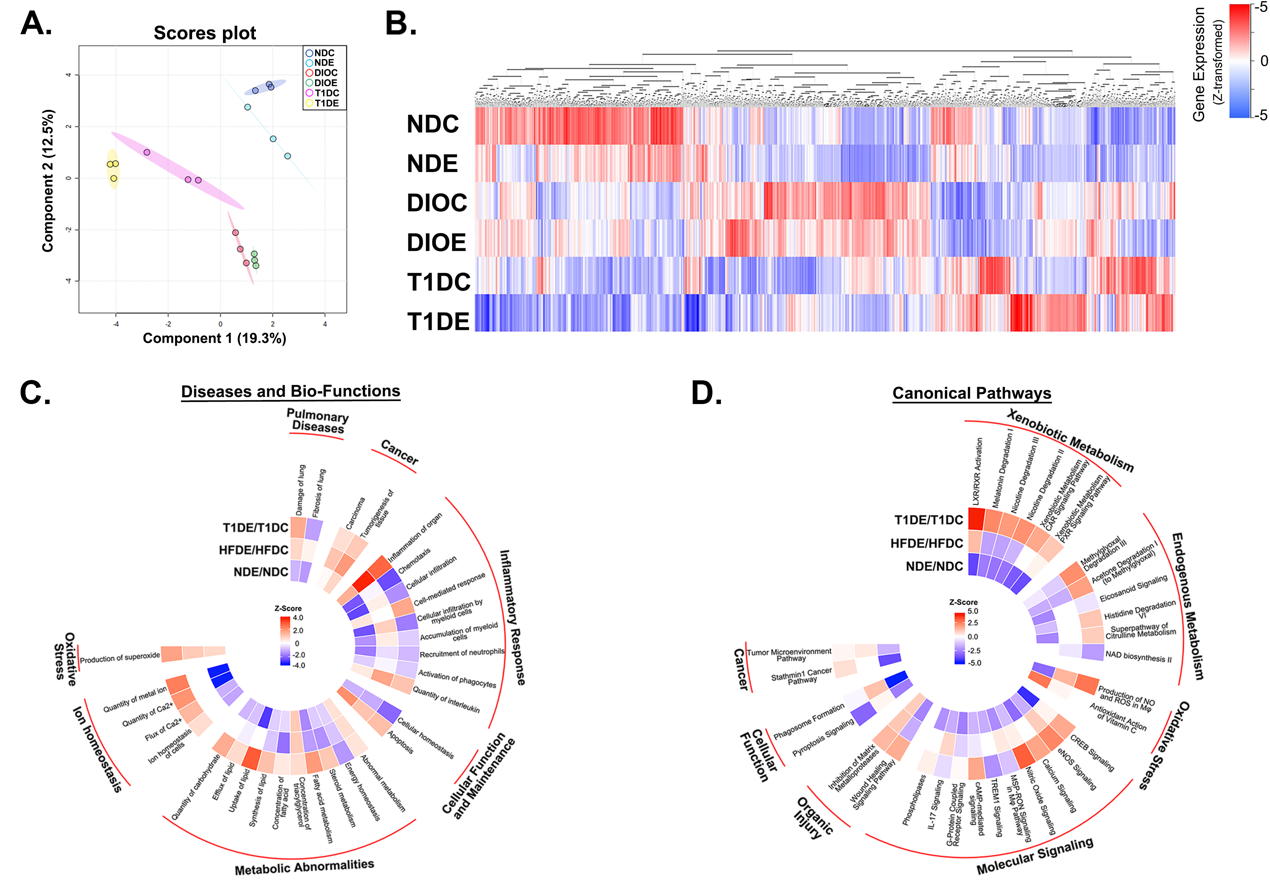


**Figure S9. Perturbations of pathways in the lungs of DIO and T1D mice exposed to PM.**

PLS-DA (A) and HCA (B) displayed overall discrimination among different groups. Lung DEGs obtained between PM exposure and control groups in DIO or T1D mouse model were introduced into IPA software for Disease and Biological Function analysis (C) and Canonical Pathway analysis (D), shown as the Circo heatmaps consisting of the top 30 enriched pathways.

**
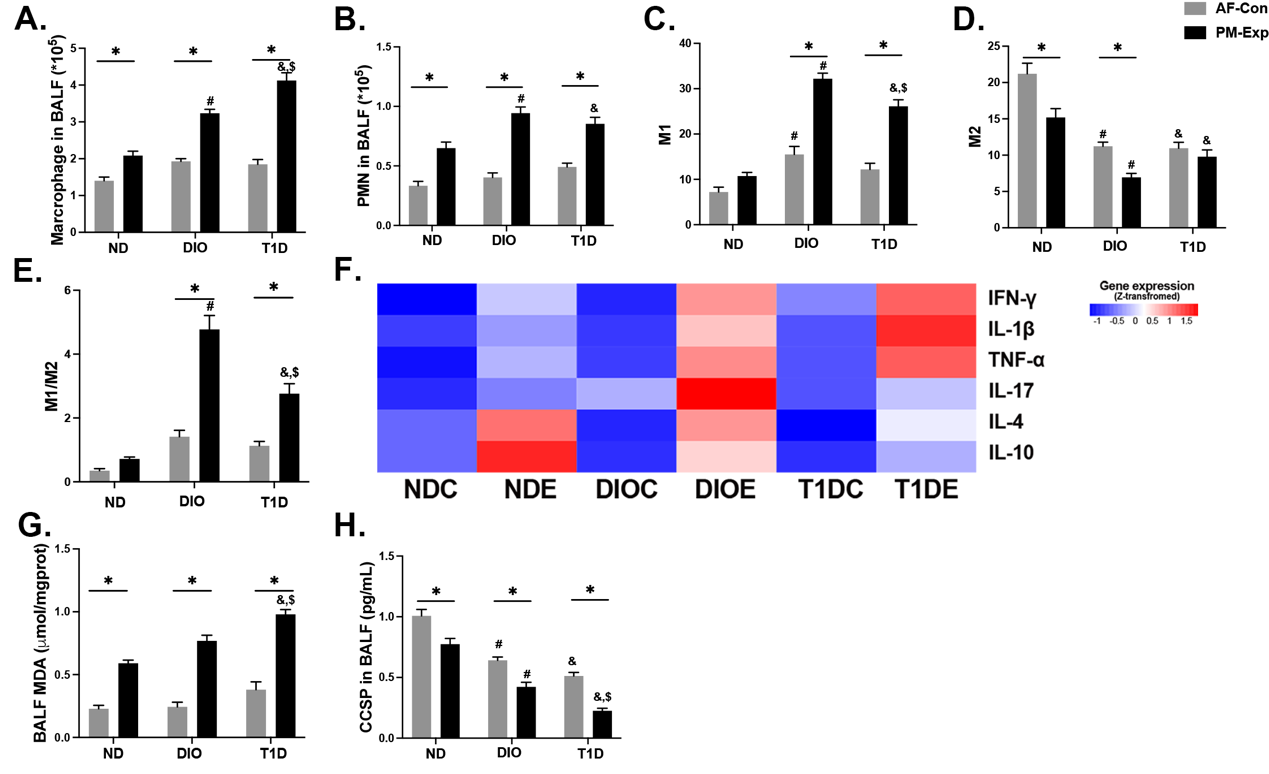
Figure S10. Key molecular events enhanced in the lungs of DIO and T1D mice upon PM exposure.**

Cell counts of macrophages (A) and polymorphonuclear cells (PMNs) (B) in BALF. Proportions of M1 (F4/80^+^/CD11b^+^/CD11c^+^/CD206^-^) macrophages (C), M2 (F4/80^+^/CD11b^+^/CD11c^-^/CD206^+^) macrophages (D), and M1/M2 ratio (E) derived from flow cytometry analysis. F. The heatmap derived from cytokines levels (IFN-γ, IL-1β, TNF-α, IL-17, IL-4, and IL-10) in BALF. G. Detection of MDA in BALF. H. The content of CCSP cytokine in BALF. *n* = 5. The data are presented as mean ± SEM. ^*^*P* < 0.05 (PM vs. AF). ^#^*P* < 0.05 (DIO vs. ND). ^&^*P* < 0.05 (T1D vs. ND). ^$^*P* < 0.05 (T1D vs. DIO).

**
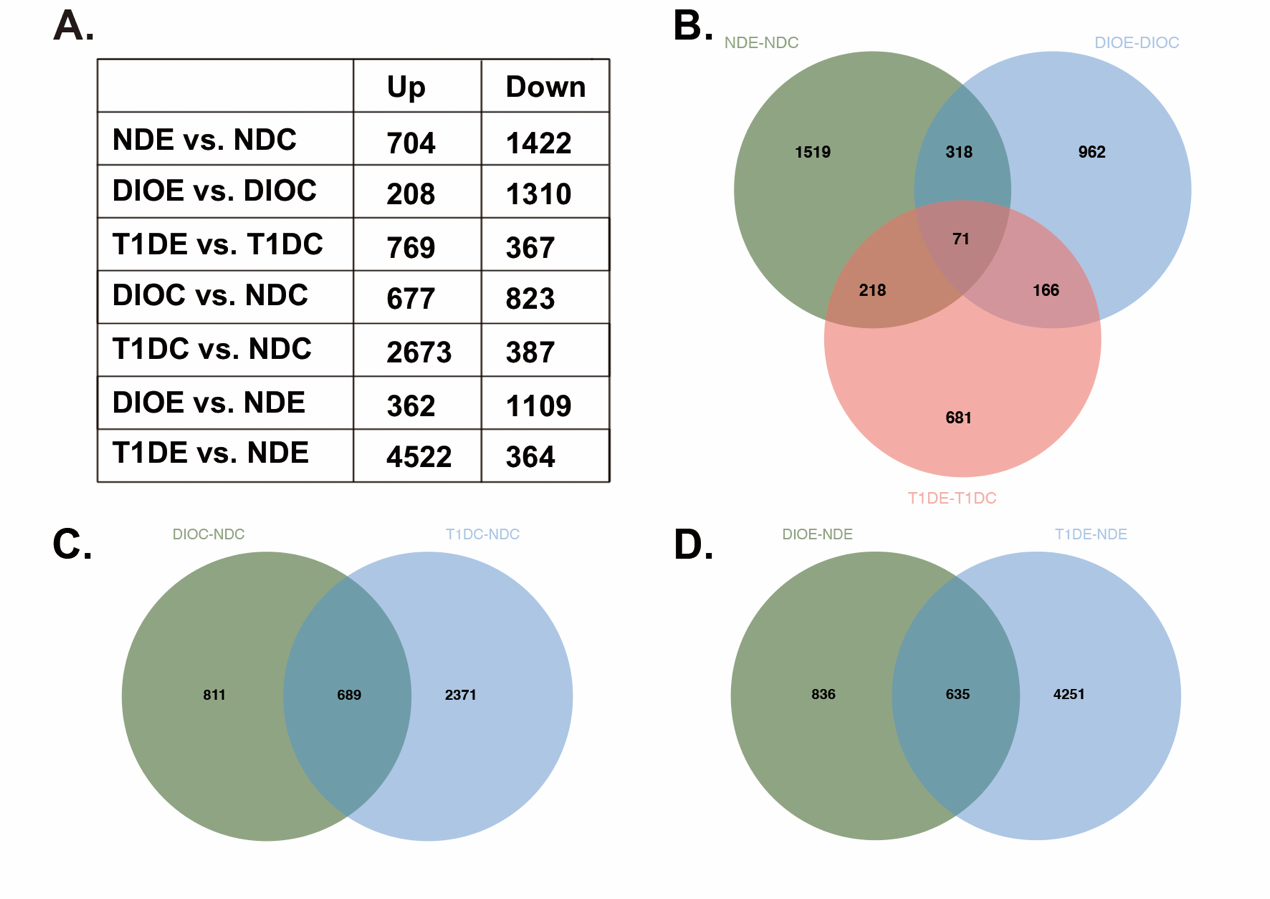
Figure S11. The multiple comparisons among differentially expressed genes (DEGs) from liver tissues.**

A. The table presents all liver DEGs for multiple comparisons. The Venn plots display the relationship among DEGs from multiple comparisons, including DEG _NDE vs. NDC_, DEG _T1DE vs. T1DC_, and DEG _DIOC vs. NDC_ (B), DEG _DIOC vs. NDC_ and DEG _T1DC vs. NDC_ (C), and DEG _DIOE vs. NDE_ and DEG _T1DE vs. NDE_ (D). *n* = 3. The threshold was set as the fold change (FC) value greater than 1.5 times, and the adjusted *P*-value less than 0.05.

**
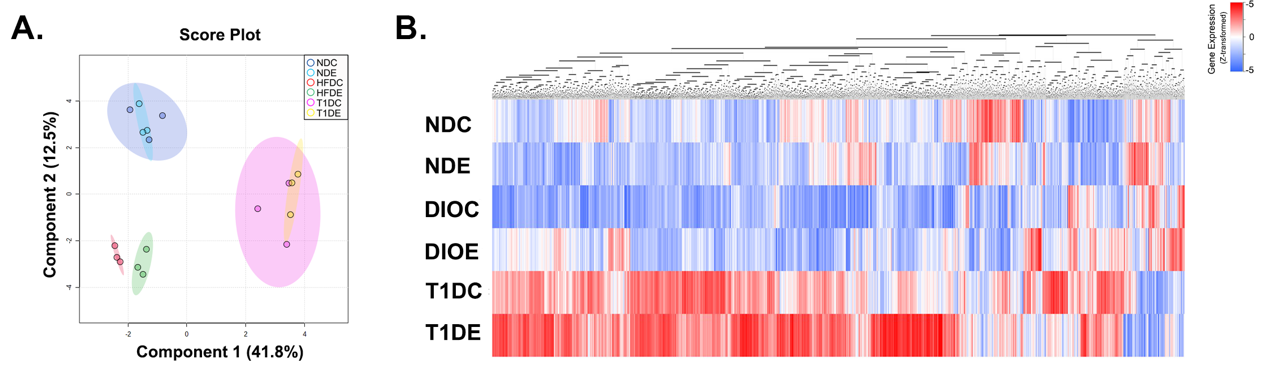
Figure S12. Differences in liver transcriptome profiles among DIO, T1D, and ND mice upon PM exposure.**

A. Partial Least-Squares Discriminant Analysis (PLS-DA). B. Hierarchical cluster analysis (HCA). *n* = 3.

**Figure S13. The quantification of protein expression from the images of protein bands.**

Aryl hydrocarbon receptor (AHR) in the lung (A) and liver (D). Constitutive androstane receptor (CAR) in the lung (B) and the liver (E). Pregnane X receptor (PXR) in the lung (C) and liver (F). *n* = 3. The data are presented as mean ± SEM. ^*^*P* < 0.05 (PM vs. AF). ^#^*P* < 0.05 (DIO vs. ND). ^&^*P* < 0.05 (T1D vs. ND). ^$^*P* < 0.05 (T1D vs. DIO).


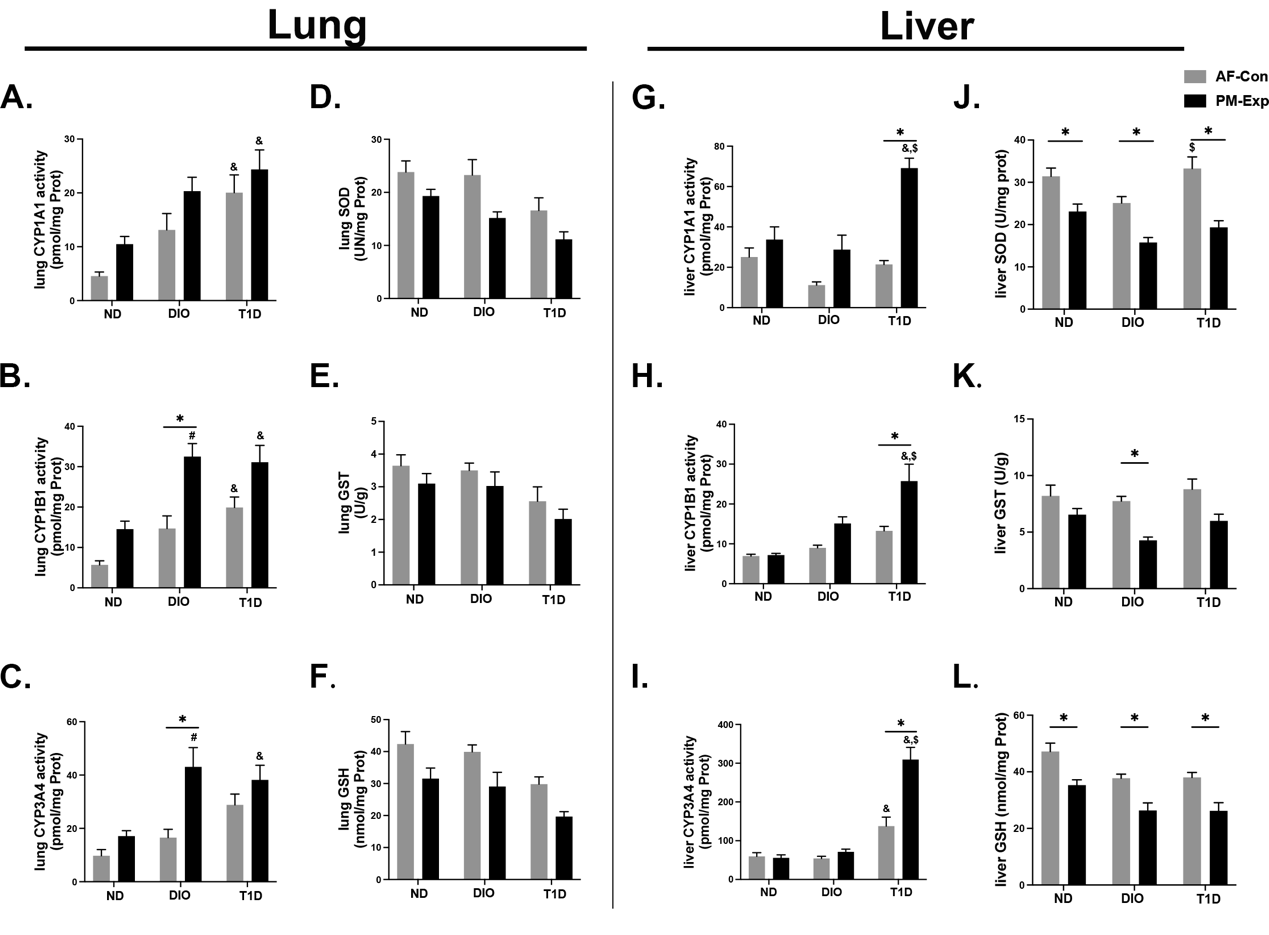


**Figure S14. Enzyme activities differentially altered in the lungs and livers of DIO and T1D mice upon PM exposure.**

Enzymatic activities of three subtypes of CYP450s, including CYP1A1 (**A, G**), CYP1B1 (**B, H**), and CYP3A4 (**C, I**) were quantified in lung and liver tissues, respectively. Detoxification ability indicated by SOD (**D, J**), GST (**E, K**), and GSH (**F, L**) was examined in mouse lungs and livers. *n* = 5. The data are presented as mean ± SEM. ^*^*P* < 0.05 (PM vs. AF). ^#^*P* < 0.05 (DIO vs. ND). ^&^*P* < 0.05 (T1D vs. ND). ^$^*P* < 0.05 (T1D vs. DIO).


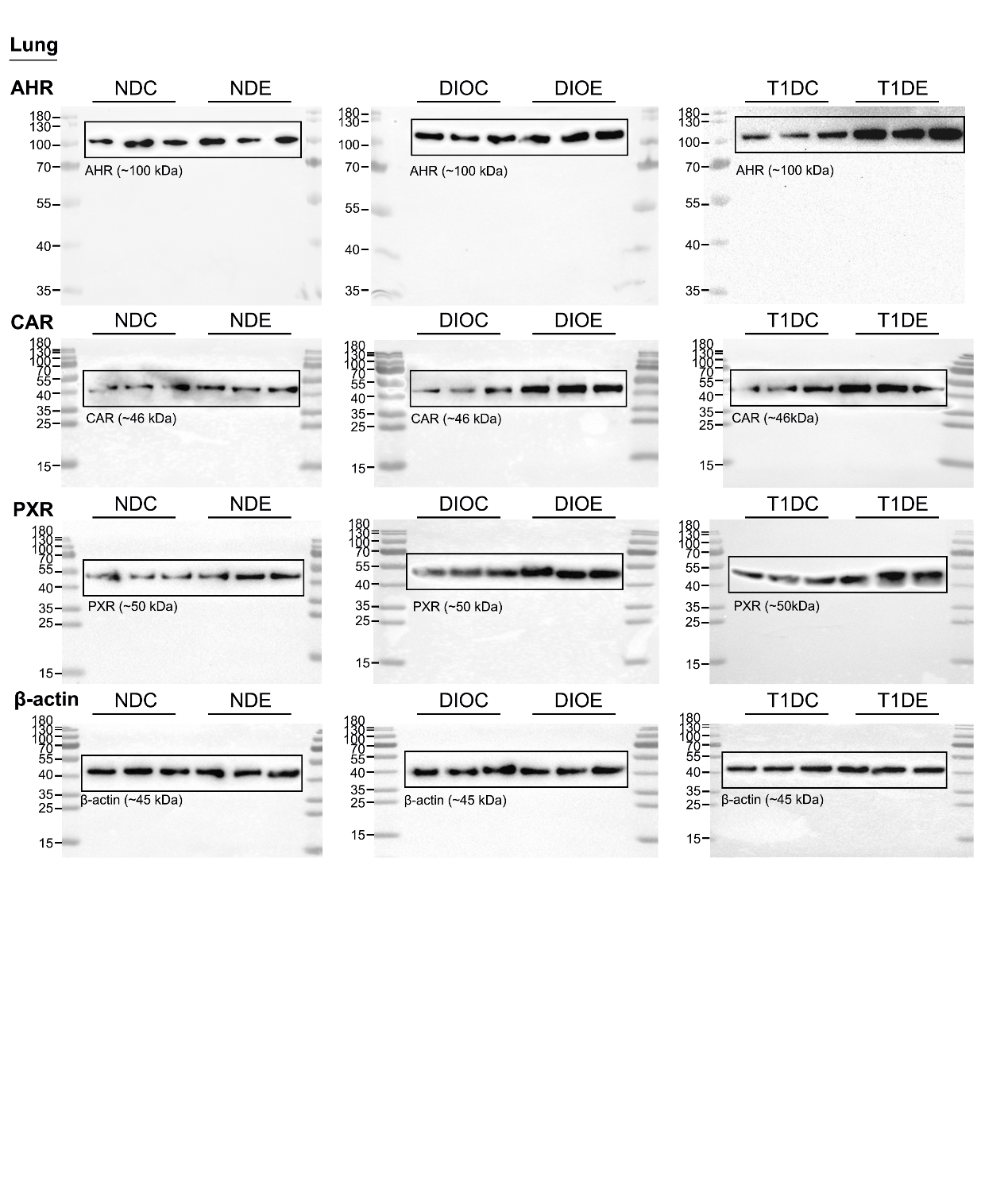


**Figure S15. The blotting images of AHR, CAR, and PXR in mouse lungs.**


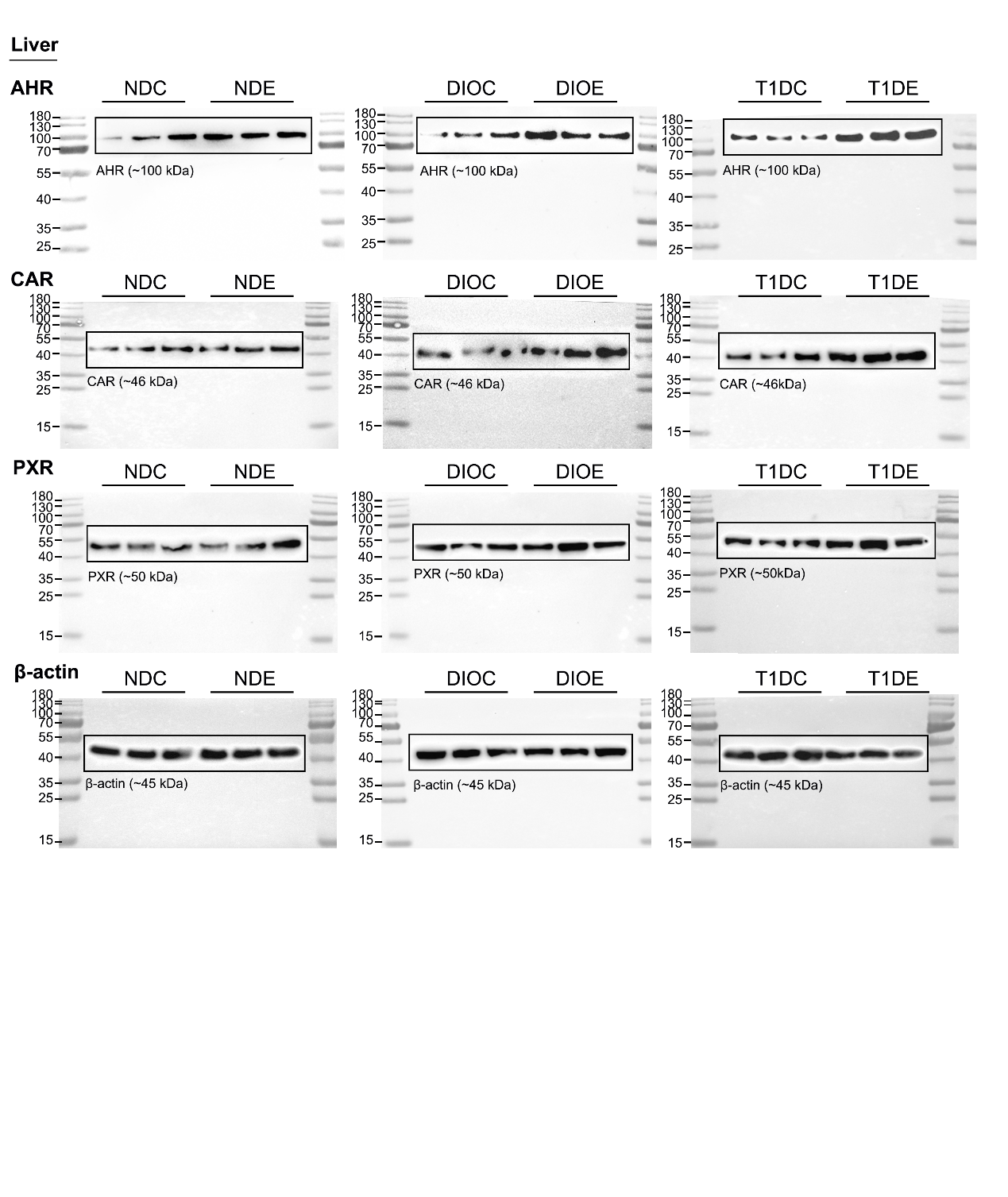


**Figure S16. The blotting images of AHR, CAR, and PXR in mouse livers.**

**Supplementary tables**

Table S1. Energy proportions of macronutrients (kcal%) in D12451 and AIN-93G diets

|  | D12451 | AIN-93G |
| --- | --- | --- |
| Protein | 20 | 18 |
| Carbohydrate | 35 | 66 |
| Fat | 45 | 16 |
| Total | 100 | 100 |

Table S2. The detailed information about the formulation of D12450 diets (4.7 Kcal/g)

| Class description | Ingredients | Contents (grams) |
| --- | --- | --- |
| Protein | Casein, Lactic, 30 Mesh | 200.00 |
| Protein | Cystine, L | 3.00 |
| Carbohydrate | Sucrose, Fine Granulated | 176.80 |
| Carbohydrate | Lodex 10 | 100.00 |
| Carbohydrate | Starch, Corn | 72.80 |
| Fiber | Solka Floc, FCC200 | 50.00 |
| Fat | Lard | 177.50 |
| Fat | Soybean Oil, USP | 25.00 |
| Mineral | S10026B | 50.00 |
| Vitamine | Choline Bitartrate | 2.00 |
| Vitamine | V10001C | 1.00 |
| Dye | Dye, Red FD&C #40, Alum. Lake 35-42% | 0.05 |
| Total: 858.15 | | |

Table S3. The detailed information about the formulation of AIN93G purified diets (3.92 Kcal/g)

| Class description | Ingredients | Contents (grams) |
| --- | --- | --- |
| Carbohydrate | Corn Starch | 397.50 |
| Protein | Casein | 200.00 |
| Carbohydrate | Maltodextrin | 132.00 |
| Carbohydrate | Sucrose | 100.00 |
| Fat | Soybean Oil | 70.00 |
| Fiber | Cellulose | 50.00 |
| Mineral | Mineral MixAIN-93G-MX | 35.00 |
| Vitamin | Vitamin MixAIN-93-VM | 10.00 |
| Protein | L-Cystine | 3.00 |
| Vitamin | Choline Bitartrate | 2.50 |
| Total: 1000.00 | | |

Table S4. The meteorological conditions in the AF control and PM exposure cages

|  | Temperature  (℃) | Humidity  （%） | Ventilation frequency  （/h) | Air-flow rate  （m/s) | Noise  （dB） |
| --- | --- | --- | --- | --- | --- |
| AF Control | 20.2±2.4 | 45.1±1.1% | 18-20 | 0.17 | 35-40 |
| PM Exposure | 21.5±3.1 | 46.3±2.1% | 18-20 | 0.17 | 35-40 |

Table S5. Primers for qRT-PCR

| Gene | Forward (5’-3’) | Reverse (5’-3’) |
| --- | --- | --- |
| **Representative components in molecular pathways (Lung)** | | |
| *Tlr1* | GCTGGTGTTAGGAGATGCTTAT | GACGGACACATCCAGAAGAAA |
| *Tlr13* | CAGAGGCCATTAGTGACATACC | CCAGAGCAGACAGATTGGTAAA |
| *Trem1* | GTCCAGTTTATCCTCTGCTCTTG | CACAGGTCACAGTCCCTTATTG |
| *Gpr35* | TCCCTTCACTTCTCCTTCCT | AGGCCTCAGGAAACTTACAATC |
| *Gpr87* | ATGACCCTGACATTCCCATTC | CAGCCCAAGAAACACGATAGA |
| *Grk1* | GACTTCTCCGTGGACTACTTTG | TGCTTCAGCTCCTTGTTCTC |
| *Grk4* | CCTGATCCTCAGGCCATTTATT | GGTATCCAGGTTGACTCCTTTC |
| *Cacng1* | CGATCTGGTCTGGTCATCTTTAG | GTTCGTATCATGGCGGAGAA |
| *Chrna1* | GTCAGACCAGGAGTCCAATAAC | CGATGAGACACACCAGCATAA |
| *Mapk10* | CTGGCTTCTCTCAGTCAGTTATC | CTATGCTCCATGACCAAGTCTC |
| *Mapk13* | TCGGAGCTTCCATGATTTCTAC | GTACACCAAGTACTGGACCTTATC |
| *Cebpb* | CTTGATGCAATCCGGATCAAAC | CCCGCAGGAACATCTTTAAGT |
| *Cebpd* | GCAGCTCCCAGAACACTAAA | TCTGTAAAGCTTCAGCCAGTATC |
| *Nlrp10* | TGAAAGAACCAGCCCTTACC | GACTTCACACCACAGGAGATAC |
| *Il1b* | ATGGGCAACCACTTACCTATTT | GTTCTAGAGAGTGCTGCCTAATG |
| *Il1r2* | CTGATAGTCCCGTGCAAAGT | GGGTAAGCAGCCGAGATAAA |
| *Il6* | GTCTGTAGCTCATTCTGCTCTG | GAAGGCAACTGGATGGAAGT |
| *Tnf* | CTACCTTGTTGCCTCCTCTTT | GAGCAGAGGTTCAGTGATGTAG |
| *Cxcl5* | TGAACTCCCTGCTTTGATGAG | CCGATAGTGTGACAGATAGGAAAG |
| *Cxcl13* | CTCGTGCCAAATGGTTACAAAG | CTTCAGGCAGCTCTTCTCTTAC |
| *Ifng* | CTCTTCCTCATGGCTGTTTCT | TTCTTCCACATCTATGCCACTT |
| *Ido1* | GCTTCTTCCTCGTCTCTCTATTG | CTTTCAGGTCTTGACGCTCTAC |
| *Ido2* | CCCTCAGACTTCCTCACTTAATC | GCTGCTCACGGTAACTCTTTA |
| *Tdo2* | CATCGTGTGGTGGTCATCTT | CTGATGCTGGAGACAGGTATTC |
| *Trp53* | CAGTCTACTTCCCGCCATAAA | GTCTCAGCCCTGAAGTCATAAG |
| *Cdkn1a* | GTTCCTTGCCACTTCTTACCT | TCATCCTAGCTGGCCTTAGA |
| *Cdkn2a* | CATGTTGTTGAGGCTAGAGAGG | CACCGTAGTTGAGCAGAAGAG |
| *Col4a1* | CTGCTCTGCGTGGAGTATTT | AGGATGAAGGAGGCTAACAAAG |
| *Col9a2* | AGCCAGGACAGAGCCTATTA | CTCCCTCACTGAACTTGTCTTC |
| *Bax* | GTGGTTGCCCTCTTCTACTTT | CAGCCCATGATGGTTCTGAT |
| **Xenobiotic metabolizing enzymes and transporters (Lung)** | | |
| *Aldh1l1* | GAGGAAGCTGGTGGAGTATTG | ACGGTTGGCTGAAAGAAGAA |
| *Aldh3a1* | CACTTCCAGCGGGTCATAAA | TAGGATGGTGGGAGCTATGT |
| *Aldh3b2* | TGGGCAGATCTGGTGACTAA | CCACCCTCTAGGTATGGATGAA |
| *Cat* | CACACACACACACATGCAATAC | TTCTGAGTGGGCCATCTTTATC |
| *Ces1b* | CTGGGCTTCTCTTGCTCTTT | CTGGTGTACTTTCCCAGGATTT |
| *Ces2e* | GCTGATGGCCTCTAAGGATTT | GGCAGGGTCTTTCTGGTTATT |
| *Ces3a* | GCCTGTCTGCTCCTGATATTT | GGATGCCCAGGAAGACATTTA |
| *Cyp1a1* | GTGAGCAAGGAGGCTAACTATC | GGCTACTGACACGACCAAATA |
| *Cyp1a2* | GTCTTCCTCTTCTTAGCCATCC | GCTTCATGGTCAACCCATAGT |
| *Cyp1b1* | TGGCCCTTTCCTCCTATCT | ACTGACACAACCTGCGTATC |
| *Cyp2a5* | GAGGAGATTGATCGGGTGATTG | CATGGATTACAGCCTCCGTATAG |
| *Cyp2e1* | GAAGTCTCTGGTTGACCCTAAG | AGGTCTCATGAACGAGGAATG |
| *Cyp3a11* | ACCACCAGTAGCACACTTTC | CCAGGTATTCCATCTCCATCAC |
| *Fmo6* | CACATCCTTCCTCCGGAATATC | CTTTCCTGAGAGGGCCATTTA |
| *Maoa* | GAAGAGAAGAACTGGTGTGAGG | CGAATCACCCTTCCATACAGAG |
| *Nqo1* | GAGAAGAGCCCTGATTGTACTG | ACCTCCCATCCTCTCTTCTT |
| *Sod3* | GAACTTCACCAGAGGGAAAGAG | CAGTAGCAAGCCGTAGAACAA |
| *Nos2* | TCTCCCTTTCCTCCCTTCTT | CTTCAGTCAGGAGGTTGAGTTT |
| *Gclc* | CATCGACCTGACCATCGATAAG | AGGGTGAGTGGGTCTCTAATAA |
| *Gsta2* | GAAGAGCCATGGACAAGACTAC | GTCAGAAGGCTGGCATCAA |
| *Gsta4* | GCTGCAGGCATTTAAGACAAG | GTCCTGACCACCTCAACATAG |
| *Sult1a1* | GATGCCCACTATGCCAAGATAA | CTTGCCTTGGTTCCCAGTATAG |
| *Sult1d1* | CAGTTCACTGTAGCCCAGTATG | CCAACTCTCCCTAGATCTCTGA |
| *Ugt1a1* | TGGACGGACTGCCTTTAATC | CAGCTAGGAGCATACTGGAATC |
| *Ugt2b1* | CTGGTGTGGCCTACAGAATAC | TGAGGATGGAAGCAGAAGATATG |
| *Abcc2* | GTGAACAGCATTCGGAAGAAAG | CAGGGTTGGAGTTAGGTGTAAG |
| *Abcc3* | GATAGCAGAGACAGGCAATGT | ACCATACAGGAGGCAGATAGA |
| *Slc18b1* | GGCTACGAGGTACCCTTTATTT | TTCCCTGGATCGGACTCATA |
| *Slc43a3* | GGGAGGAATCCTGTTCCTTATC | TGCTGAGGAGGAGTCAAATG |
| *Slc4a4* | CTCAAGAAAGGAGCAGGGTATC | GAGCCATGAGGGAGCATATAAC |
| **Representative components in molecular pathways (Liver)** | | |
| *Atf3* | CTCCTGGGTCACTGGTATTTG | CCGATGGCAGAGGTGTTTAT |
| *Atf4* | CCACTCCAGAGCATTCCTTTAG | CTCCTTTACACATGGAGGGATTAG |
| *Cybb* | ATGAGTTCCACACCTTCCTTC | GGCTTGAGACAACCTGGTATTA |
| *Fos* | GAATCCGAAGGGAACGGAATAA | TCTCCGCTTGGAGTGTATCT |
| *Cebpd* | GCAGCTCCCAGAACACTAAA | TCTGTAAAGCTTCAGCCAGTATC |
| *Mafk* | TTGGGCCTTCTTGTCCTTATC | ACACACAGGAACACAGACATTA |
| *Map2k6* | GGATACGGGCCACAGTTAATAG | GTAGAAGGTCACGGTGAATGG |
| *Map4k3* | GTACGTGGCTGTATGTGATGAA | CCGGTAAGTTGTGGGAGTAAAG |
| *Mapk7* | GTGAGGCTTGGCTTGAGTTA | GTAGAGTCTCCTGGTCCTGTTA |
| *Nfe2l2* | CTCCGTGGAGTCTTCCATTTAC | GCACTATCTAGCTCCTCCATTTC |
| *Stat3* | GCCACGTTGGTGTTTCATAATC | TTCGAAGGTTGTGCTGATAGAG |
| *Id1* | AGGTGAACGTCCTGCTCTA | GATCTCCACCTTGCTCACTTT |
| *Traf2* | CAGCCTTCTACACAAGCAGATA | CCACGAAGAAGAGAGACAGATG |
| *Hspa1b* | TGGTGCTGACGAAGATGAAG | CGCTGAGAGTCGTTGAAGTAG |
| *Hspa8* | CCCTTCATGGTGGTGAATGA | GACACTTCCTCTGGGTAGAAAC |
| *Il1a* | GCTTGAGTCGGCAAAGAAATC | GAGAGATGGTCAATGGCAGAA |
| *Il1b* | ATGGGCAACCACTTACCTATTT | GTTCTAGAGAGTGCTGCCTAATG |
| *Il1r1* | AGGTGGAGGACTCAGGATATT | CCAGGGTCATTCTCTAACACAG |
| *Ccl2* | CTCGGACTGTGATGCCTTAAT | TGGATCCACACCTTGCATTTA |
| *Casp1* | TACACGTCTTGCCCTCATTATC | CTCCAGCAGCAACTTCATTTC |
| *Cdkn1a* | GTTCCTTGCCACTTCTTACCT | TCATCCTAGCTGGCCTTAGA |
| *Bax* | GTGGTTGCCCTCTTCTACTTT | CAGCCCATGATGGTTCTGAT |
| *Bcl2* | GAGCAGGTGCCTACAAGAAA | CTTTGTCCTCTGACTGGGTATG |
| *Gadd45b* | CCTGGTCACGAACTGTCATAC | GGTTATTGCCTCTGCTCTCTT |
| *Gadd45g* | TCCGCCAAAGTCCTGAATG | CGCCTGAATCAACGTGAAATG |
| **Xenobiotic metabolizing enzymes and transporters (Liver)** | | |
| *Aldh2* | CTGGACCAGTGTGTGCTTATT | CCTTGGACTTGACCTGCTTATC |
| *Ces1g* | CATGGAGGTGGACTTGTGATAG | GGCGATACTGAATGACCACTAC |
| *Cyp1a1* | GTGAGCAAGGAGGCTAACTATC | GGCTACTGACACGACCAAATA |
| *Cyp1a2* | GTCTTCCTCTTCTTAGCCATCC | GCTTCATGGTCAACCCATAGT |
| *Cyp2e1* | GAAGTCTCTGGTTGACCCTAAG | AGGTCTCATGAACGAGGAATG |
| *Cyp2a5* | GAGGAGATTGATCGGGTGATTG | CATGGATTACAGCCTCCGTATAG |
| *Cyp3a11* | ACCACCAGTAGCACACTTTC | CCAGGTATTCCATCTCCATCAC |
| *Fos* | GAATCCGAAGGGAACGGAATAA | TCTCCGCTTGGAGTGTATCT |
| *Nqo1* | GAGAAGAGCCCTGATTGTACTG | ACCTCCCATCCTCTCTTCTT |
| *Gclc* | CATCGACCTGACCATCGATAAG | AGGGTGAGTGGGTCTCTAATAA |
| *Gsta1* | GTGAGCTGAGTGGAGAAGAAG | CCGGGCATTGAAGTAGTGAA |
| *Gstm1* | CTCACGCTTCCTAGAATTACCC | CAGGCTGGCACTCAAGTATT |
| *Gstm2* | AAGCCTCAGCTACCCACTAT | CAACCACTAACAGGAAGGAAGG |
| *Gstm3* | GAGGATCCGTGTGGATACTTTG | GAACTCTGGCTTCTGCTTCT |
| *Gstm5* | CCCACAGCGTCCAGTATAAA | CCAGTAACCCAGAACCATAGAC |
| *Gstp1* | GAGACCTCACCCTTTACCAATC | CCCATCATTCACCATATCCATCT |
| *Gstt3* | GAGTGGAGGGAAAGAAAGTGAA | GAGGAACGGAACCCAGTAAAG |
| *Mgst3* | GTACAGCACAGATCCTGAGAAC | GTAAACACCTCCCACCGTTAG |
| *Sult1a1* | GATGCCCACTATGCCAAGATAA | CTTGCCTTGGTTCCCAGTATAG |
| *Sult1b1* | ACTATGCTCCCTGGCTATCT | CCAGCCTGGTCTACACTATTTC |
| *Sult1d1* | CAGTTCACTGTAGCCCAGTATG | CCAACTCTCCCTAGATCTCTGA |
| *Sult2a8* | TCTCTCCTTCCCATCCAACTA | TAGCCTGACACAAGAACATCTC |
| *Ugt1a1* | TGGACGGACTGCCTTTAATC | CAGCTAGGAGCATACTGGAATC |
| *Ugt1a9* | CCTCTTTAGCCCAGTGTCTATTT | GGCAGTTGATCCCACCAATA |
| *Abcc3* | GATAGCAGAGACAGGCAATGT | ACCATACAGGAGGCAGATAGA |
| *Abce1* | GAAGTCCAGCAGATCCCTAAAG | GCCAGAGTGTCTTCCTCAATTA |
| *Abcd1* | CTCTCGGATCACATGAAGGAAG | GTCTTCAGGGAATGCAGGTAA |
| *Slc1a2* | TCTGTCTCTGTCTCTCCCTTT | CTGGTTCCTGGAGTTAGTGTATG |
| *Slc2a2* | CTTACAGTCACACCAGCATACA | AGACAGAGACCAGAGCATAGT |
| *Slc2a1* | AAGACTGCTGCTCAGATCTATTC | GGAGATAGGAGAGTGGCTGATA |
